# Transmission of stony coral tissue loss disease (SCTLD) in simulated ballast water confirms the potential for ship-born spread

**DOI:** 10.1038/s41598-022-21868-z

**Published:** 2022-11-10

**Authors:** Michael S. Studivan, Michelle Baptist, Vanessa Molina, Scott Riley, Matthew First, Nash Soderberg, Ewelina Rubin, Ashley Rossin, Daniel M. Holstein, Ian C. Enochs

**Affiliations:** 1 Cooperative Institute for Marine and Atmospheric Studies, University of Miami, 4600 Rickenbacker Causeway, Miami, FL 33149 USA; 2 NOAA Atlantic Oceanographic and Meteorological Laboratory, Ocean Chemistry and Ecosystems Division, 4301 Rickenbacker Causeway, Miami, FL 33149 USA; 3 Rosenstiel School of Marine and Atmospheric Science, University of Miami, 4600 Rickenbacker Causeway, Miami, FL 33149 USA; 4 Excet, Inc., 6225 Brandon Ave #360, Springfield, VA 22150 USA; 5 U.S. Naval Research Laboratory, 4555 Overlook Ave SW, Washington, DC 20375 USA; 6 Department of Oceanography and Coastal Sciences, College of the Coast and Environment, Louisiana State University, Baton Rouge, LA 70803 USA; 7 Present Address: University of Florida, 2033 Mowry Rd, Gainesville, FL 32611 USA

**Keywords:** Microbiology techniques, Biofilms, Microbial communities, Environmental microbiology, Infectious-disease diagnostics, Pathogens, Ecological epidemiology, Tropical ecology

## Abstract

Stony coral tissue loss disease (SCTLD) remains an unprecedented epizootic disease, representing a substantial threat to the persistence and health of coral reef ecosystems in the Tropical Western Atlantic since its first observation near Miami, Florida in 2014. In addition to transport between adjacent reefs indicative of waterborne pathogen(s) dispersing on ocean currents, it has spread throughout the Caribbean to geographically- and oceanographically-isolated reefs, in a manner suggestive of ship and ballast water transmission. Here we evaluate the potential for waterborne transmission of SCTLD including via simulated ballast water, and test the efficacy of commonly-used UV radiation treatment of ballast water. Two species of reef-building corals (*Orbicella faveolata* and *Pseudodiploria strigosa*) were subjected to (1) disease-exposed or UV-treated disease-exposed water, and (2) a ballast hold time series of disease-exposed water in two carefully-controlled experiments to evaluate transmission. Our experiments demonstrated transmission of SCTLD through water, rather than direct contact between diseased and healthy corals. While UV treatment of disease-exposed water led to a 50% reduction in the number of corals exhibiting disease signs in both species, the statistical risk of transmission and volume of water needed to elicit SCTLD lesions remained similar to untreated disease-exposed water. The ballast hold time (24 h vs. 120 h) did not have a significant effect on the onset of visible disease signs for either species, though there appeared to be some evidence of a concentration effect for *P. strigosa* as lesions were only observed after the 120 h ballast hold time. Results from both experiments suggest that the SCTLD pathogens can persist in both untreated and UV-treated ballast water and remain pathogenic. Ballast water may indeed pose a threat to the continued spread and persistence of SCTLD, warranting further investigation of additional ballast water treatments and pathogen detection methods.

## Introduction

An unprecedented outbreak of stony coral tissue loss disease (SCTLD) has continued largely unabated in the Tropical Western Atlantic since 2014. This disease is known to affect at least 24 scleractinian coral species and is characterized by rapid onset of disease lesions, leading to tissue loss and colony mortality over a period of days to weeks^1–4^. To date, a pathogen has not been identified, but there is evidence of bacterial involvement due to effectiveness of antibiotic treatments^5–7^. Alternatively, viral presence in disease-affected coral tissues and algal endosymbiont cells^8,9^ and potential coinfections of microbial taxa^10–16^ also support the potential for a pathogenic microbial consortium. It has been suggested through local hydrodynamic modeling and ex situ experiments that SCTLD is likely transmitted via water^3,15,17–21^, with additional suspected modes of transmission through biotic (e.g., butterfly fish)^22^ and abiotic sources (e.g., sediments, ballast water)^10–12,23,24^.

Since its first observation near Miami, Florida in 2014, SCTLD has spread throughout the entirety of Florida’s Coral Reef and to numerous jurisdictions in the Caribbean, including Jamaica, Mexico, St. Maarten, U.S. Virgin Islands, Dominican Republic, and Belize^2,25^. The initiation of SCTLD outbreaks in very distant locations suggests that disease transport has been aided by means other than dispersal on ocean currents, such as through ship ballast water and biofilms in ballast systems, as ships take on water in a region with epidemic or endemic disease and release it in a naïve port^12,23,24^. Through an examination of the proximity of commercial ports to observations of SCTLD from 2014–2020, Rosenau et al.^24^ hypothesized a potential link between the two, particularly for geographically- or oceanographically-isolated reefs. In the Bahamas, Dahlgren et al.^23^ reported that new observations of SCTLD occurred rapidly from late 2019 to early 2020 in close proximity to larger cities and ship discharges. Similarly in the U.S. Virgin Islands, initial SCTLD observations were seen closer to human centers^26^.

Although the relationship between coral disease outbreaks and ballast water transfer has not been extensively studied, ships are known to serve as pathways for the introduction of non-native marine species and pathogens, both for commercial vessels^27,28^, as well as smaller recreational and fishing vessels^29,30^. Ship ballast water, associated particulates, and biofilms in the ballast system and ships’ wetted surfaces can host a diverse array of microorganisms, including pathogens and parasites^31–35^. For example, a study examining ballast water microbial communities in commercial vessels in the southern Gulf of Mexico identified high concentrations of coliforms frequently associated with ‘white plague’ coral diseases such as *Vibrio cholerae*^36^. While it has not been empirically confirmed, the unprecedented rapid spread of white band disease in the late 1970s was suspected to be the result of a pathogen introduction via the Panama Canal or ballast water transfers in the region^37^.

**Figure 1:**
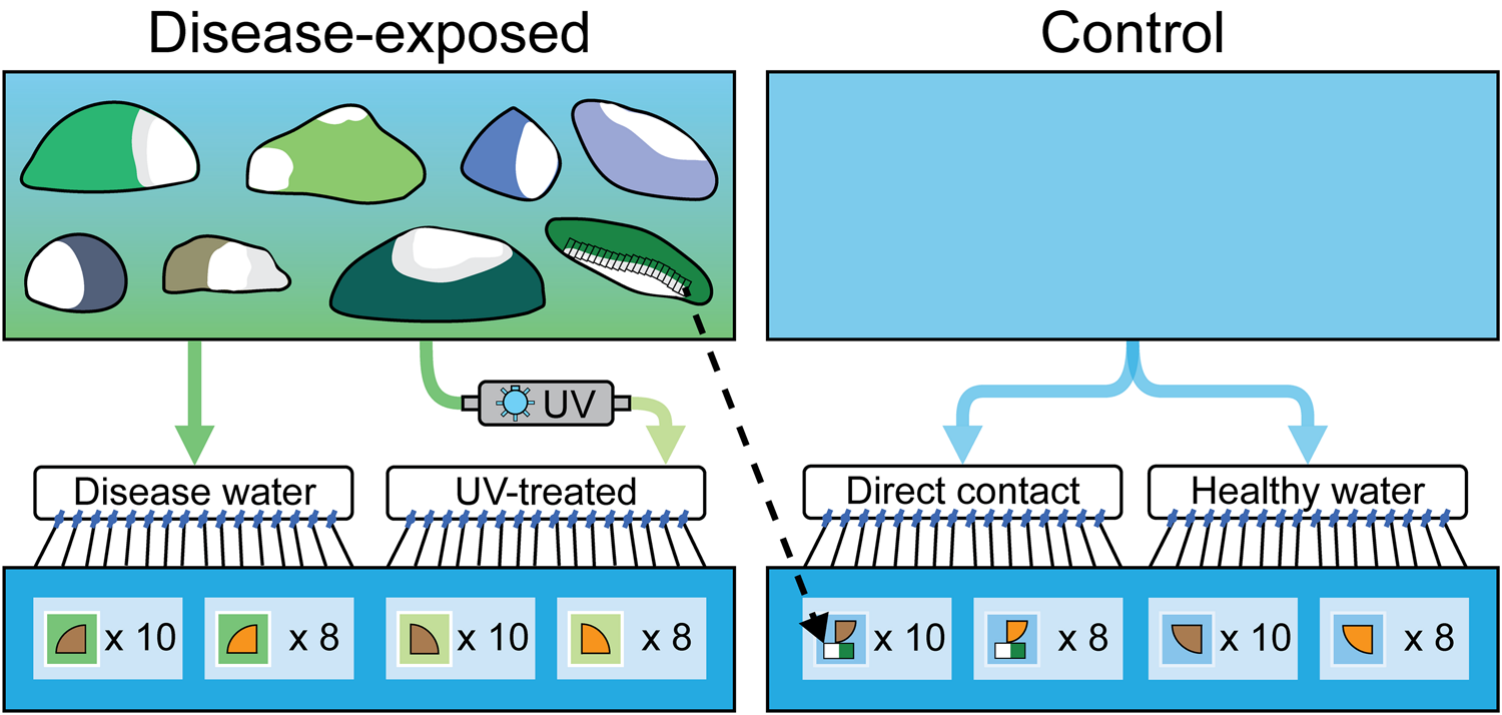
UV experiment infographic. SCTLD transmission apparatus in the Experimental Reef Laboratory at the University of Miami’s Cooperative Institute for Marine and Atmospheric Studies. Disease water generation using field-collected colonies of *Montastraea cavernosa* exhibiting SCTLD lesions (top), subsequent separation of water treatments and in-line UV treatment using Sanitron S17A 3-GPM UV system (middle), and exposure of water treatments to randomized fragments of *Orbicella faveolata* (brown) and *Pseudodiploria strigosa* (orange) in individual 0.5 L vessels with independent water sources from manifold systems (bottom).

**Figure 2:**
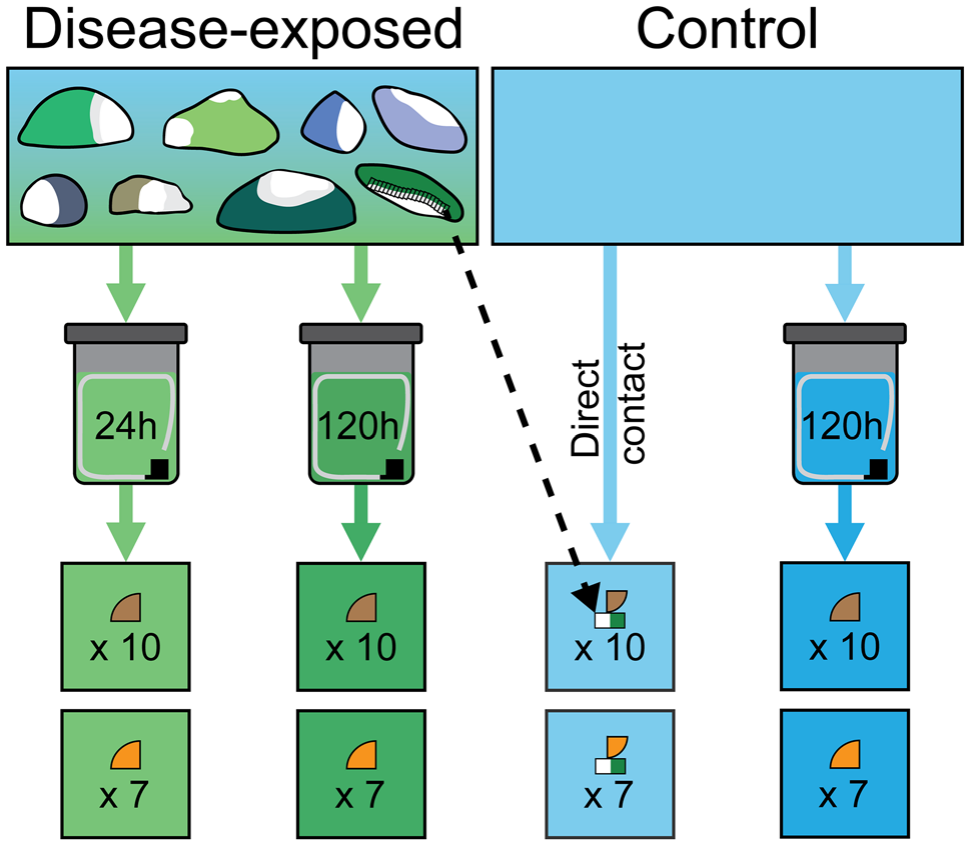
Ballast water experiment infographic. Disease water generation using field-collected colonies of *Montastraea cavernosa* exhibiting SCTLD lesions (top), subsequent ballasting in sealed 208 L (55 gal) containers (middle), and exposure of water treatments to fragments of *Orbicella faveolata* (brown) and *Pseudodiploria strigosa* (orange) in 150 L aquaria via daily water changes over seven days (bottom).

**Figure 3:**
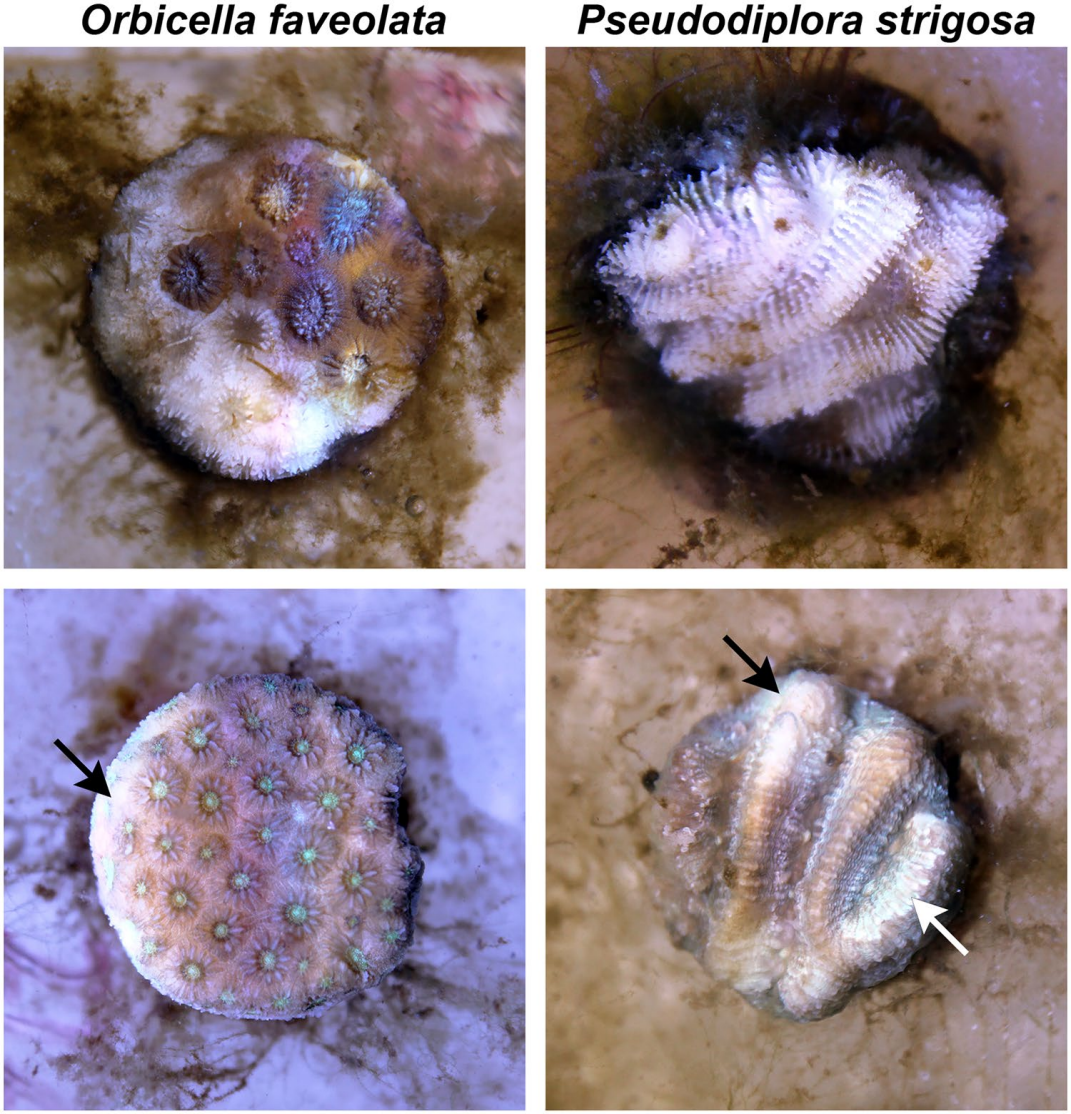
Representative photos of disease lesions. *Orbicella faveolata* (left) and *P. strigosa* (right) fragments following contact with disease-exposed water. *Orbicella faveolata* fragments typically exhibited rapid onset of necrosis/tissue loss following initial exposure to SCTLD (top left) and/or paling/bleaching margins (bottom left, indicated by arrow). *Pseudodiploria strigosa* fragments demonstrated rapid (< 24 h) progression of tissue loss once lesions were first observed (top right), with less frequent occurrence of paling/bleaching lesions (bottom right, indicated by arrows).

The potential for SCTLD transport via ships, therefore, has numerous practical management implications. For example, ballast water transfers can be regulated in local, federal, and international jurisdictions, and typically require some level of treatment prior to release in local waters (reviewed in Rosenau et al.^24^). Common ballast water management systems, or BWMS, may include mechanical filtration, UV radiation, chlorination, ozonation, or some combination of multiple treatment methods^24,38^. Experimentally evaluating waterborne transport, the effectiveness of ballast water treatment, and the impacts of ballast water hold time are therefore necessary for informed action and disease mitigation strategies. Using an experimental approach, we sought to test three hypotheses that are fundamental to the potential spread and treatment of SCTLD via ballast water: 1) transmission risk following contact with disease-exposed water can be reduced using UV radiation (i.e., a common method in BWMS; Fig. 1), 2) simulated ballasting of disease-exposed water affects infectiousness over time (Fig. 2), and 3) established ballast water testing protocols can detect the presence of SCTLD in disease-exposed water.

## Results

### Waterborne disease transmission and UV treatment

**Figure 4:**
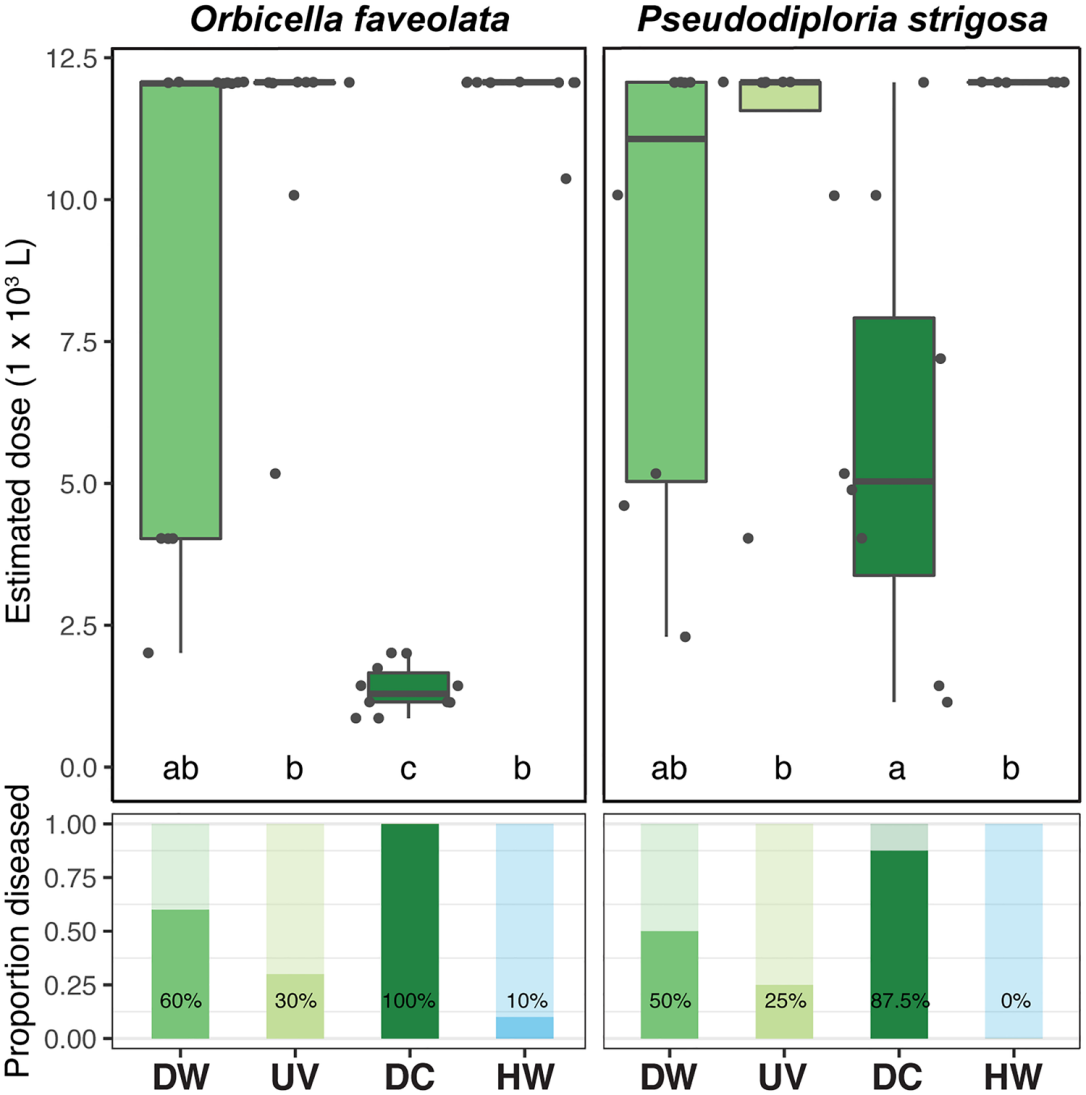
UV experiment transmission metrics. Estimated water dose ± SEM in liters needed to elicit SCTLD lesions (boxplots) and transmission rates (proportion of individuals exhibiting lesions). Colors denote treatments, and different letters denote significant differences among treatments. Treatment abbreviations are as follows: disease water (DW), UV-treated disease water (UV), diseased coral direct contact (DC), and healthy water (HW).

**Figure 5:**
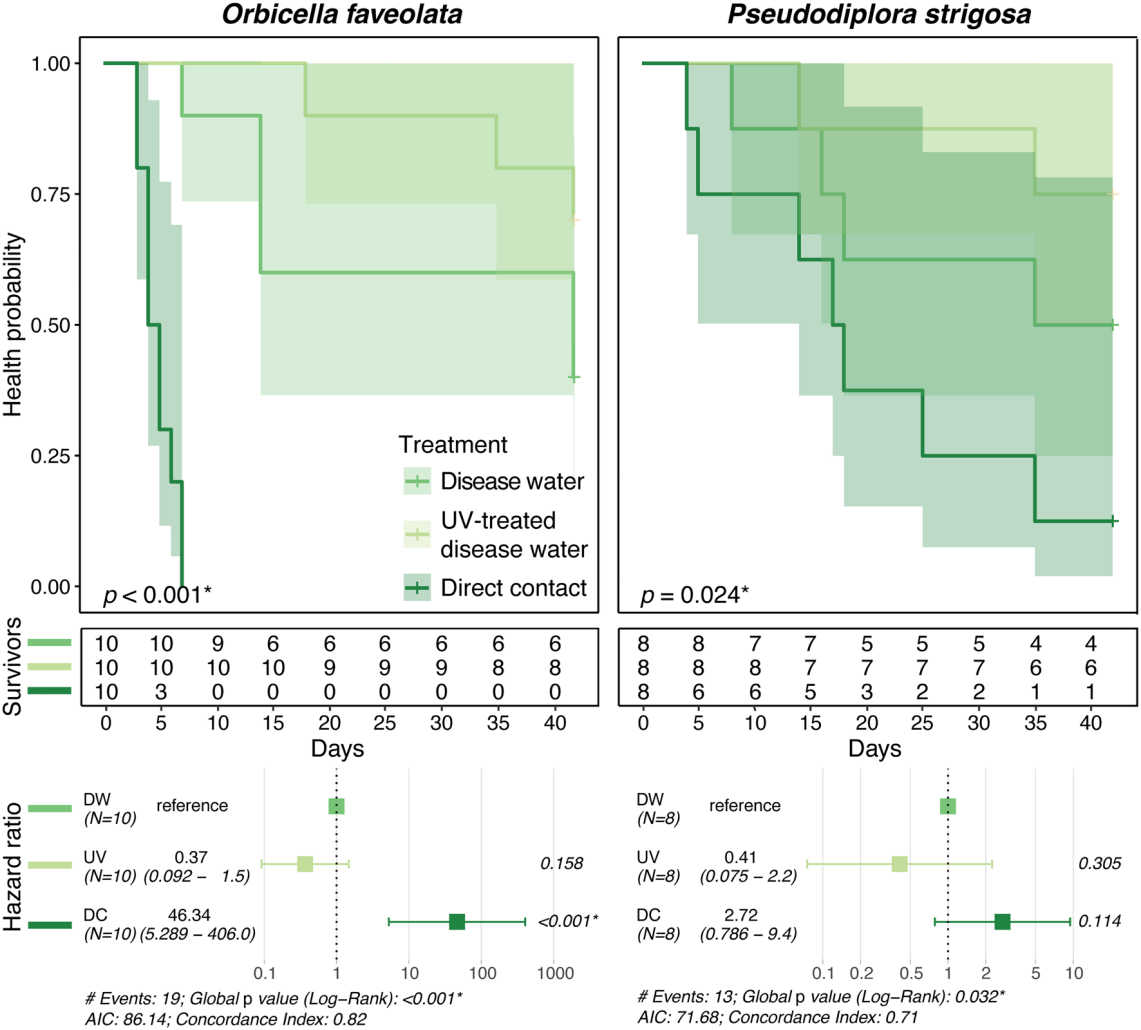
UV experiment survivorship curves. Mean time to initial observations of SCTLD lesions and transmission rates (proportion of individuals exhibiting lesions), with survivorship tables and hazard ratio tests of infection risk between treatments. Colors denote treatments, shaded areas denote 95% CI, and test statistics reflect the results of fit proportional hazards regression models for each species. Treatment abbreviations are as follows: disease water (DW), UV-treated disease water (UV), diseased coral direct contact (DC), and healthy water (HW).

Visible signs of SCTLD (e.g., paling/bleaching, lesion formation, rapid tissue loss; Fig. 3) were observed in 60% of *Orbicella faveolata* and 50% of *Pseudodiploria strigosa* fragments in the untreated disease water treatment, with the elicitation of visible lesions occurring after 22.1 ± 6.3 days and 19.2 ± 5.7 days, respectively (Table 1). UV treatment of disease-exposed water corresponded to a 50% reduction in the proportion of individuals exhibiting disease signs for both species (30% and 25%, respectively), with slightly longer but more variable times to onset of lesions (31.6 ± 7.1 days for *O. faveolata* and 24.5 ± 10.5 days for *P. strigosa*). In the disease contact (disease control) treatment, disease signs were observed for 100% of *O. faveolata* and 87.5% of *P. strigosa* fragments, with visible lesions forming earlier on average for both species (4.8 ± 0.5 days and 16.8 ± 4.1 days, respectively). One individual of *O. faveolata* in the healthy control treatment was observed to have potential signs of SCTLD occurring near the end of the experiment after 36 days, which was suspected to be accidental contamination. When time to onset of lesion data were used to calculate the respective volume of water exposed to treatments (i.e., water ‘dose’), there was a significant effect of water treatments on water dose (ANOVA: *F*_3,71_ = 35.144, *p* < 0.001), with pairwise tests attributing significant variation between the disease contact treatment and all other treatments, and between healthy water and disease water treatments (all *p* < 0.01; Fig. 4; Supplementary Table S1). While water dose was not applicable to lesion formation for the disease contact treatment, the metric was used as a proxy for time to onset of lesions for significance testing. Risk of lesion formation was not significantly different between untreated disease water and UV-treated disease water treatments for both species. There was, however, a significant difference between untreated disease water and disease contact treatments for *O. faveolata* only (log-rank: *z*_2,29_ = 3.464, *p* < 0.001; Fig. 5).

**Table 1:** UV experiment transmission metrics.

Species	Treatment	Treatment Abbrev	Sample count	Lesion count	Transmission rate (%)	Time to lesion (d)	SEM (d)	Estimated dose (L)	SEM (L)
*Orbicella faveolata*	Disease water	DW	10	6	60.0	22.1	6.3	8646	4447
	UV-treated disease water	UV	10	3	30.0	31.6	7.1	11,178	2198
	Disease contact	DC	10	10	100.0	4.8	0.5	1379	426
	Healthy water	HW	10	1	10.0	36.0		11,897	539
*Pseudodiploria strigosa*	Disease water	DW	8	4	50.0	19.2	5.7	8804	4094
	UV-treated disease water	UV	8	2	25.0	24.5	10.5	10,814	2829
	Disease contact	DC	8	7	87.5	16.8	4.1	5753	3864
	Healthy water	HW	8	0	0.0			12,067	0

Transmission rates (proportion of individuals exhibiting SCTLD lesions), mean ± SEM time to onset of lesions in days, and estimated water dose ± SEM in liters needed to elicit disease lesions.

### Ballast water disease transmission

**Figure 6:**
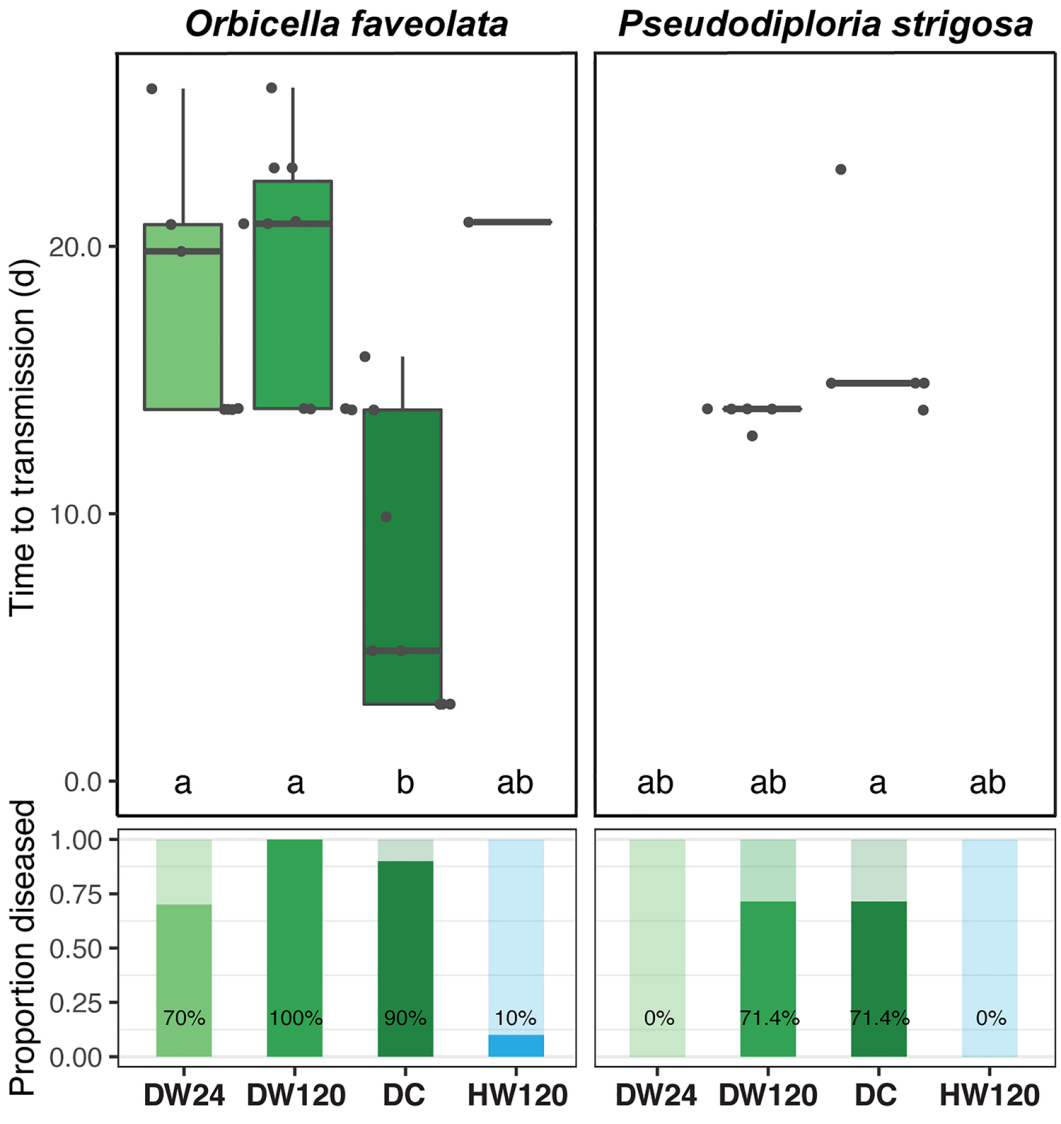
Ballast experiment transmission metrics. Mean time to initial observations of SCTLD lesions ± SEM (boxplots) and transmission rates (proportion of individuals exhibiting lesions). Colors denote treatments, and different letters denote significant differences among treatments. Treatment abbreviations are as follows: disease water 24 h (DW24), disease water 120 h (DW120), diseased coral direct contact (DC), and healthy water 120 h (HW120).

**Figure 7:**
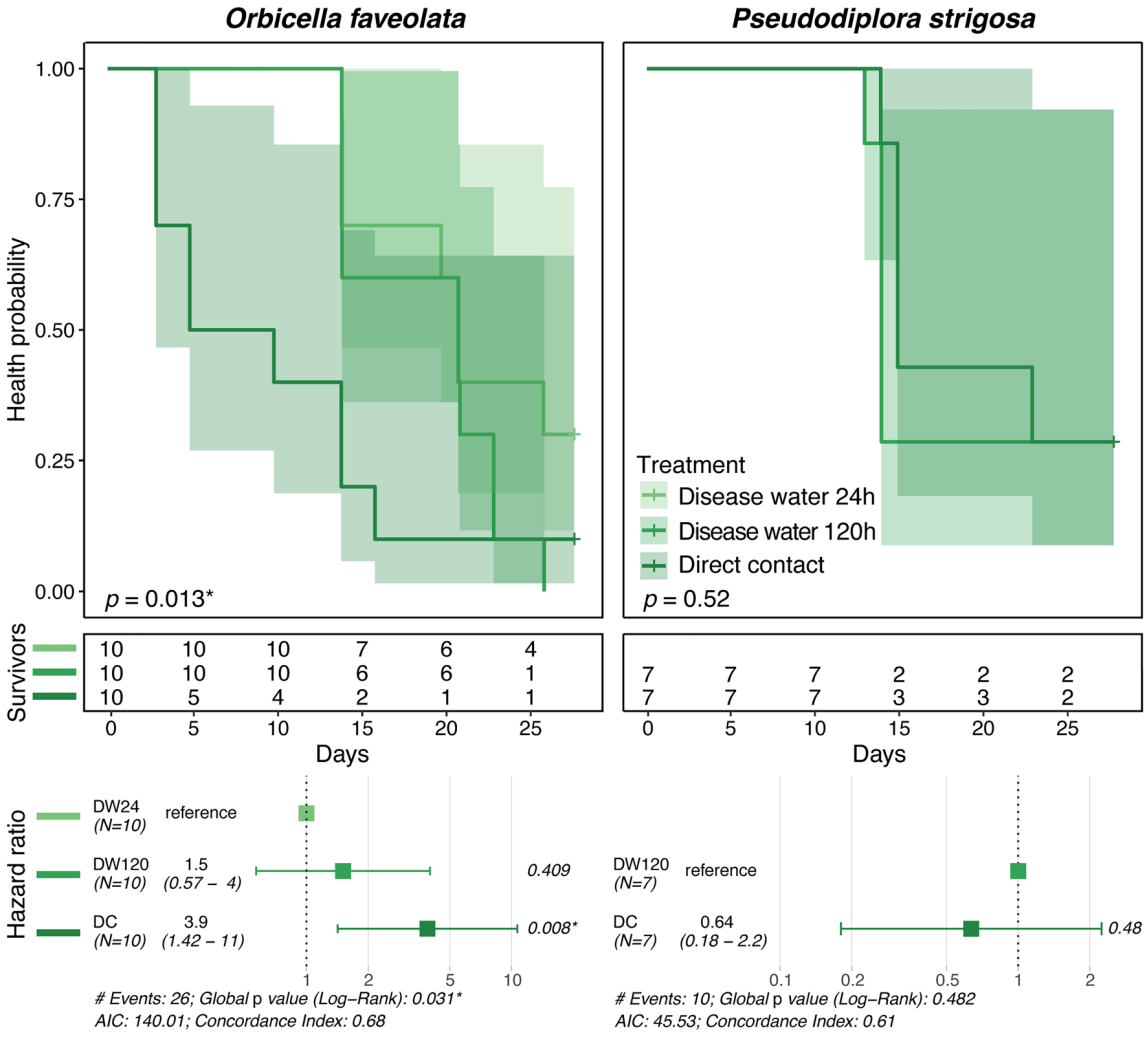
Ballast experiment survivorship curves. Mean time to initial observations of SCTLD lesions and transmission rates (proportion of individuals exhibiting lesions), with survivorship tables and hazard ratio tests of infection risk between treatments. Colors denote treatments, shaded areas denote 95% CI, and test statistics reflect the results of fit proportional hazards regression models for each species. Treatment abbreviations are as follows: disease water 24 h (DW24), disease water 120 h (DW120), diseased coral direct contact (DC), and healthy water 120 h (HW120).

Following contact with disease-exposed water that had a ballast hold time of 24 h, no transmission was reported for *P. strigosa*, but 70% of *O. faveolata* exhibited signs of disease, with visible lesions forming after 18.4 ± 1.8 days in the latter (Table 2). Signs of SCTLD were observed in both species following contact with disease-exposed water with a hold time of 120 h, with 100% of *O. faveolata* fragments (19.0 ± 1.5 days) and 71.4% of *P. strigosa* fragments (13.7 ± 0.2 days). SCTLD lesions were observed in 90% of *O. faveolata* and 71.4% of *P. strigosa* fragments in the disease contact (disease control) treatment, with the onset of visible lesions occurring after 8.0 ± 1.8 days and 16.3 ± 1.7 days, respectively. One individual of *O. faveolata* in the healthy control treatment was observed to have signs consistent with SCTLD occurring after 21 days, which was likely due to inadvertent contamination as it was observed near the end of the experiment. There was a significant effect of water treatments on time to onset of disease lesions (ANOVA: *F*_3,36_ = 7.449, *p* < 0.001), with pairwise tests attributing significant variation between the disease contact treatment and both disease ballast treatments for *O. faveolata* only (both *p* < 0.006; Fig. 6; Supplementary Table S2). Risk of lesion formation was not significantly different between ballast water hold time treatments for *O. faveolata*, but there was a significant decrease in risk with exposure to ballast water held for 24 h compared to the disease contact treatment (log-rank: *z*_2,29_ = 2.642, *p* < 0.009). Pairwise comparisons between hold time treatments were not possible for *P. strigosa*, as no individuals in the 24 h hold time treatment demonstrated disease signs, however, there was not a significant difference in relative risk between 120 h hold time and disease contact treatments (Fig. 7).

**Table 2:** Ballast experiment transmission metrics.

Species	Treatment	Treatment Abbrev	Sample count	Lesion count	Transmission rate (%)	Time to lesion (d)	SEM (d)
*Orbicella faveolata*	Disease water 24 h	DW24	10	7	70.0	18.4	1.8
	Disease water 120 h	DW120	10	10	100.0	19.0	1.5
	Disease contact	DC	10	9	90.0	8.0	1.8
	Healthy water 120 h	HW120	10	1	10.0	20.9	
*Pseudodiploria strigosa*	Disease water 24 h	DW24	7	0	0.0		
	Disease water 120 h	DW120	7	5	71.4	13.7	0.2
	Disease contact	DC	7	5	71.4	16.3	1.7
	Healthy water 120 h	HW120	7	0	0.0		

Transmission rates (proportion of individuals exhibiting SCTLD lesions) and mean ± SEM time to onset of lesions.

### Histological analysis

Disease signs were detected in 93% of disease-exposed samples across both experiments. Additionally, 41% of healthy control samples analyzed histopathologically showed signs of stress, dysbiosis, and/or disease (Supplementary Table S3). In addition to liquefactive necrosis, vacuolization of symbionts, exocytosis, and gastrodermal separation, coral samples determined to be diseased occasionally showed loss of eosin stain from the mesoglea (typical of cellular lysing), pyknotic nuclei in symbiont cells, and disruption of internal tissue structures. These results imply that histological identification of SCTLD may be muddled by artifacts of coral tissue health status from long-term husbandry in ex situ aquarium settings.

### Ballast water analysis

There was no significant effect of water treatments on total live eukaryotic cell counts for the UV experiment, but counts varied significantly through time (ANOVA; *F*_3,35_ = 69.614, *p* < 0.001), with all pairwise comparisons significant among time points except for week 2 versus week 4 (Tukey; all other *p* < 0.004). Heterotrophic bacterial concentrations were highly variable among treatments and through time, with a minimum of ≤ 2.1 × 10^2^ to a maximum of > 1.1 × 10^5^ colony forming units (CFU) mL^-1^ with no detectable or predictable pattern (Supplementary Table S5). In the ballast experiment, total eukaryotic live cell counts were significantly different among treatments and time points with a significant interaction effect (ANOVA; time: *F*_1,17_ = 4.895, *p* < 0.047; treatment: *F*_2,17_ = 12.540, *p* < 0.001; interaction: *F*_2,17_ = 6.433, *p* < 0.013; Supplementary Table S6), however there was no discernable pattern among treatments. Likewise, there were no notable differences in heterotrophic bacterial concentrations among treatments, though concentrations ranged from ~ 10^5^ to > 10^6^ CFU mL^-1^ following the initial ballasting period, then increased across all treatments to ranges near or above the limit of detection after an additional week of ballasting (Supplementary Table S7).

## Discussion

These data corroborate that SCTLD can be transported through water, without requiring direct contact between diseased and healthy corals^3,17–20^ (and references therein). SCTLD transmission was significantly less likely to occur via water than from coral-to-coral (see also Aeby et al.^3^), highlighting the importance of more accurately simulating transmission dynamics in future lab-based experiments. Application of in-line UV radiation resulted in a 50% reduction in the number of individuals exhibiting disease signs for both coral species tested, however, significance testing using relative risk analysis indicated that UV treatment of disease-exposed water did not result in a significant reduction in risk of lesion formation for either species (Figs. 4, 5). While actual UV dosages used by BWMS are often confidential (e.g., see^39^), the dosage used in this study (50 mWs^-1^ cm^-2^, equivalent to 500 Jm^-2^) is comparable to that used in other studies^40^. These results imply that UV treatment does not significantly mitigate the overall risk of developing SCTLD lesions over time compared to untreated disease-exposed water.

There was also evidence that ballasting increased the infectiousness of disease-exposed water over time. There were observed SCTLD lesions in over 70% of *P. strigosa* fragments in the disease-exposed ballast water held for 120 h treatment, however, no lesions were observed when exposed to disease-exposed ballast water held for 24 h, suggesting that a ‘concentration effect’ may be occurring over several days of ballast hold time. Similarly, there was an increase in transmission rates for *O. faveolata* exposed to ballast water held for 120 h versus 24 h (Fig. 6), corroborating that longer ballast hold time may increase the relative risk of SCTLD infection (Fig. 7). It is likely that the impacts of pathogen concentration following ballast hold times are species-specific, given that SCTLD susceptibility and signs vary among affected coral taxa^2,3,15,17^. Similarly, pathogen abundance may continue to change at hold times greater than 120 h (such as during a long voyage), potentially due to prolonged periods in aphotic environments and depleting resources. Examination of microbial communities in disease-exposed ballast water is warranted to determine if microbial communities, particularly SCTLD-associated microbes^10–14,16^, shift in their composition or abundance during the ballasting process, as has been demonstrated with previous studies of other microbial taxa^41,42^ including with known pathogens^43^. Continued investigation into SCTLD pathogens and co-infecting taxa^10–13,16^ in abiotic media (i.e., disease-exposed water and sediments) may identify putative pathogens, as well as determine precise bacterial testing (bioindicators) required to reflect changes in bacterial concentrations in relation to disease prevalence.

### Implications for ballast water management and treatment

Results from these experiments imply that ballast water may pose a threat to the continued persistence and spread of SCTLD throughout the Caribbean, and perhaps to coral reefs in the Indo-Pacific. The Panama Canal serves as a major trade route connecting the Atlantic and Pacific oceans^44,45^, and increasing shipping traffic and ballast water transfers on both sides of the canal have been predicted to lead to rising occurrence of species introductions^46,47^ including potential pathogens. Given the broad susceptibility of Caribbean coral species to SCTLD^2,3^, and the recent hypothesis that the SCTLD pathogen may be a virus affecting algal symbionts of the family Symbiodiniaceae^8^, Indo-Pacific corals (and/or their symbionts) could also be susceptible to SCTLD. It is therefore of critical importance to mitigate the potential risk of SCTLD transport via ships’ ballast water, as it may represent an important contributing factor for this disease to spread across ocean basins. There are many inherent factors that affect the ability of ballast water to transport pathogens, however, including the proximity of ballast water transfers to coral populations and disease-affected individuals, pathogen load and potential concentration in ballast systems, and duration of ballast hold times. Additional experiments and investigations of ballast exchange records are therefore necessary, particularly those that simulate the size and scale of ship transport parameters and ballast systems, to determine the risk associated with ballast water transfers in transporting coral disease pathogens.

UV radiation, which is a commonly-used ballast water treatment, will likely not be successful in mitigating disease spread through ballast water. Additional treatments found commonly in BWMS such as filtration, chlorination, and ozonation (reviewed in^38^), are likely to be more effective means of reducing the risk of SCTLD spread through ships’ ballast water as they have shown to have strong biocidal properties^48^, with a lower potential of bacterial regrowth^49,50^ or UV resistance^51–53^ as has been reported with UV treatment alone. There are, however, logistical and cost limitations associated with the implementation and maintenance of more sophisticated BWMS on ships, including considerations for additional fuel usage and cost–benefit biological risk assessments associated with discharge impacts with untreated versus treated ballast water^54–57^. Further, chemical treatments require neutralization and/or removal of byproducts prior to release^38,58,59^, posing potential challenges for ship-based ballast management, as well as evaluation of treatment effectiveness against SCTLD in lab-scale experimental approaches.

Water testing conducted according to established ballast water testing standards^60^ revealed few consistent patterns across treatments for both experiments. The incorporation of ballast water testing into disease exposure challenges in this study identified a disconnect between established ballast water metrics and the risk of disease transmission, where ballast water testing is typically focused on quantifying eukaryotic plankton and culturable heterotrophic bacteria. There is therefore a need to develop more appropriate ballast water standards to include potential coral pathogens including SCTLD-associated microbes, to enable rapid detection and prevention of disease introductions on reefs throughout the Caribbean and Indo-Pacific regions. Metagenomic, metatranscriptomic, and fractionation approaches are likely to be particularly useful in isolating and identifying SCTLD-indicator taxa in suspected disease sources^9,10,61^ such as ballast water, and in evaluations of treatment approaches through quantitative assessments of microbial abundance (*sensu*^62^). Combined with evaluations of transmission risk and treatment effectiveness with ballast water sources, development of coral disease bioindicators is necessary for effective ballast water mitigation and policy to ensure that applicable national and international biosecurity requirements sufficiently address coral disease mitigation strategies from ballast water and/or biofilm sources^24^. These strategies are essential to our response to the ongoing SCTLD epizootic as well as future disease outbreaks, as they directly impact our ability to curb disease spread among the Caribbean, and especially to potentially vulnerable coral reef communities in the Indo-Pacific.

UV treatment alone is likely not effective in stopping the spread of SCTLD via ballast water. This has profound implications for the treatment and management of ballast water transfers throughout the Caribbean endemic zone and suggests that enhanced monitoring and management are needed to quantify and mitigate the risk of further disease spread through human-mediated transport. Other BWMS treatment approaches, or combinations of multiple treatments, may be more effective in halting SCTLD transport through water, though these approaches currently remain untested. The United States Coast Guard released a Marine Safety Information Bulletin relevant to the SCTLD outbreak^24,63^ to reinforce existing guidelines related to ballast water exchanges that may reduce the potential for shipborne disease spread, however, targeted research on the persistence of SCTLD pathogens in ballast systems is recommended to be investigated and implemented for effective management of disease spread. Ship-based transport is not likely to decrease in the future, and our ability to react to this coral disease epidemic, as well as our ability to prevent and/or mitigate future disease outbreaks, is contingent on comprehensive management and enforcement of human-mediated pathogen sources.

## Methods

Two ex situ disease transmission experiments were conducted in the Experimental Reef Laboratory (ERL) at the University of Miami’s Cooperative Institute for Marine and Atmospheric Studies (CIMAS). Colonies of the coral species *Orbicella faveolata* and *Pseudodiploria strigosa*, both of which are characterized as susceptible to SCTLD^2^, were sourced from local reef sites near Miami, Florida. Coral colonies were split into fragments of equal size (~ 5 cm^2^), with sets of four fragments from the parent colony used for each of the treatment groups in the respective experiments. Fragments were allowed to acclimate for six months in the lab, and were considered healthy as no signs of SCTLD were observed during this time.

### Waterborne transmission and UV treatment

Fragments of ten *O. faveolata* and eight *P. strigosa* unique colonies were used per four treatments (*N* = 72 samples total): ‘healthy water’ (water not exposed to corals), ‘disease water’ (water exposed to diseased corals), ‘UV-treated disease water’ (water exposed to diseased corals, then passed through a UV water treatment system), and ‘diseased coral direct transmission’ (diseased corals directly touching apparently healthy corals; Fig. 1). Corals in the direct transmission treatment group were used to confirm that the disease donor colonies were indeed capable of transmitting SCTLD. The experimental apparatus is described in full in Studivan et al.^12^. Briefly, coral fragments were independently housed in 0.5 L vessels with flow-through water sources, and suspended in raceways using a custom-built apparatus to ensure consistent environmental parameters and to minimize disease transmission risk among vessels. Tank temperatures were maintained at 29 °C based on local reef ambient conditions at the time of the experiments.

Field collections were conducted on July 2, 2021 in Broward County, Florida (26.1479, − 80.0939) to harvest eight coral colonies of the species *Montastraea cavernosa* exhibiting visible lesions of SCTLD for disease water generation and disease donor fragments. Small fragments (~ 2 × 3 cm) of one of the disease donor colonies were cut with a diamond bandsaw for each of the experimental corals in the direct transmission treatments for the respective experiments. Direct contact was maintained between disease donor and experimental coral fragments over the course of the experiments, and donor fragments were replaced as needed following total tissue loss and donor fragment mortality. ‘Healthy’ and ‘disease water’ were generated in separate 250 L raceways using flow-through water inputs pre-filtered to 25 μm, with the SCTLD-exhibiting *M. cavernosa* coral colonies in the disease-exposed raceway. Manifold systems were then used to divide ‘disease water’ treatments into non-treated and UV-treated water supplies for downstream coral fragments.

The process of determining UV dose is described in the Supplementary Information. Briefly, UV treatment was achieved using an in-line Sanitron UV chamber (Atlantic Ultraviolet Corporation, Hauppauge, NY) equipped with a low-pressure mercury bulb that generated germicidal UV as water flowed through the chamber. The dose–response of cultured bacteria (*Escherichia coli*) was measured by exposing dilute suspensions of bacteria to UV light from a custom-built, collimated beam light source, where UV dosage was calculated using UV fluence (mW cm^-2^) and exposure time following established protocols^64^. Effective dosage of the Sanitron UV chamber was determined using a standard curve of flow rates (i.e., residence time in the chamber, pertaining to UV dosage) to bacterial concentrations, where a flow rate of approximately 11.4 L min^-1^ (~ 3 gal min^-1^) was equivalent to 50 mWs^-1^ cm^-2^ (Supplementary Fig. S1). Flow rate during the disease challenge experiment was monitored was an in-line flow meter.

The UV experiment was conducted for six weeks, with daily monitoring of individual corals for disease signs. Numerous steps were taken to minimize the spread of disease among treatment groups (e.g., fully redundant environmental monitoring equipment, sterilization of handling tools in between submersion in treatment tanks, personal protective equipment). Following observation of SCTLD lesions in experimental corals, individual coral fragments were preserved in 10% zinc-buffered formalin for tissue histology. Corals not showing any signs of SCTLD were preserved in the same fashion at the end of the experiment. Disease transmission data were analyzed by first quantifying the number of days between initial exposure to disease treatments and visible disease signs (e.g., white lesions, tissue loss). In order to analyze the time to lesions data without influence from missing values (i.e., if a fragment did not have visible disease signs by the end of the experiment), ‘water dose’ (i.e., volume in L) needed to initiate visible signs was calculated for all treatments using the constant flow rate (0.2 L min^-1^) multiplied by the number of days until initial signs, or total days in the experiment for corals not exhibiting disease signs.

### Ballast water disease transmission

Ten *O. faveolata* and seven *P. strigosa* colony fragments were used per four treatments (*N* = 68 samples total). ‘Healthy’ and ‘disease-exposed’ water was collected from the disease transmission apparatus 16 days after the start of the UV experiment. Water was subsequently held in 208 L (55 gal) containers with lids to simulate a ship ballast tank for 24 h and 120 h based on conventional ballast water holding standards (Fig. 2)^60^. Corals in the healthy control treatment were exposed to ballast water held for 120 h that had no prior exposure to corals. Corals in a direct transmission treatment were exposed to the same unexposed water from the UV experiment without any prior ballasting, but with the addition of disease donor fragments of *M. cavernosa* generated as described above. Corals in two disease water treatments were exposed to ballast water held for 24 h and 120 h following contact with entire disease donor *M. cavernosa* colonies as described for the UV experiment. All coral fragments were housed in communal 150 L aquaria (two aquaria per treatment group) with independent recirculating sump pumps and water quality monitoring instruments (aquarium systems fully described in^65^), for a total of eight aquaria. Each tank had a corresponding 208 L (55 gal) ballast container, with the exception of the direct transmission treatment tanks which used non-ballast water sourced directly from the ‘healthy water’ manifold in the UV experiment. Ballast water was oxygenated using an air stone and mixed using a submersible pump for 1 h before exposure to the communal aquaria with an initial application of 50% of each ballast container’s water by volume, and then daily 10% water exchanges for a total of 7 days (chosen based on initial lesion observations reported in previous experiments^12,17^). Following the seven-day ballast water exposures, each tank received fresh seawater from independent valves at a rate of 0.2 L min^-1^ for the remaining duration of the experiment. The ballast experiment was conducted for four weeks, with experimental corals monitored following the methodologies described for the UV experiment.

### Statistical analysis

Data were collected and analyzed in the same manner from the respective experiments to maximize comparability among UV and ballast datasets. All statistical analyses were conducted in the *R* statistical environment^66^. Both experiment datasets did not meet the assumptions for parametric analyses, and were transformed using a Box-Cox transformation prior to subsequent tests. Two-way ANOVAs were conducted across species and treatments for the respective datasets, with pairwise Tukey’s tests of significant factors. For both experiments, the transmission rate was calculated as the proportion of fragments exhibiting disease signs within each treatment. To quantify the relative risk of developing lesions among disease treatments, a survivorship analysis was conducted for each experiment using the time to lesion formation and transmission rate data using the *R* package *survival*^67^ and *survminer*^68^ for visualization. A fit proportional hazards regression model was applied to compare risk (hazard ratios) between disease treatments using the disease water (DW) treatment as a reference for the UV experiment, and using the disease-exposed ballast water held for 24 h (DW-24) treatment as a reference for the ballast experiment. Healthy control treatment data were removed prior to the survivorship analysis for both experiments, as disease transmission was not expected for these treatments.

### Histological analysis

Histological examination was conducted on a subset of coral fragments in both experiments to determine if coral tissues displayed signs consistent with SCTLD. Two to four replicates per treatment were compared depending on suitability of fragments for histological tissue preparation, for a total *n* = 24 samples for the UV experiment and *n* = 18 samples for the ballast experiment. Sample processing was conducted as described in Studivan et al.^12^, but in short, samples were first decalcified using 1% EDTA HCl solution. Tissue areas that excluded obvious lesions were further processed using a Leica ASP6025 tissue processor, embedded in paraffin wax blocks on a Leica EG1150H embedding machine, and sectioned on a Leica RM2125RTS microtome, with slides stained with hematoxylin and eosin on a Leica ST5020. Slides were analyzed for disease signs on an Olympus BX41 microscope with a SC180 camera attachment. Five serial slides, separated by ~ 500 μm, were reviewed for disease signs per individual coral sample. Slides were read blinded, and scored for presence or absence of disease signs. The presence of disease was considered confirmed when liquefactive necrosis (LN) along the basal body wall (BBW), vacuolization of symbionts, exocytosis, and gastrodermal separation^4,12,15^ were consistent across five serial histopathology sections per individual.

### Ballast water analysis

Water samples were collected weekly from each of the water treatments (healthy, disease, and UV-treated disease water) for the first four weeks of the UV experiment, with a final sampling at six weeks. In the ballast experiment, samples were collected from ballast containers corresponding to the healthy water 120 h, disease water 24 h, and disease water 120 h treatments following the ballasting period and following the seven-day water change period. Samples were processed and analyzed according to established ballast water testing protocols^60,69,70^, and are described in full in the Supplementary Information. Briefly, live cell counts (10–50 μm, nominally protists) were conducted using an epifluorescence microscope and a combination of the vital fluorophores, chloromethylfluorescein diacetate (CMFDA) and fluorescein diacetate (FDA)^71^. Data were square-root transformed and analyzed for variation among time and treatments using a two-way ANOVA with Tukey’s pairwise tests of significant factors; multivariate variation was assessed using PERMANOVAs in the packages *vegan* and *pairwiseAdonis*^72,73^. Heterotrophic bacteria were quantified (most probable number [MPN] of colony forming units [CFUs]) using heterotrophic plate counts (HPC) on IDEXX SimPlates (IDEXX; Westbrook, ME) as described by the manufacturer.

## Supplementary Information

Supplementary Information.

## Data Availability

Datasets generated from this study can be found in the Supplementary Materials, and analysis scripts can be found in a GitHub repository release^74^.
