# Supplementary material for: Transmission of stony coral tissue loss disease (SCTLD) in simulated ballast water confirms the potential for ship-born spread
Source: Sci Rep. 2022 Nov 10;12:19248. doi: 10.1038/s41598-022-21868-z (PMC9649619; doi:10.1038/s41598-022-21868-z)
Supplement: Supplementary file 1 — Supplementary Information. [file 41598_2022_21868_MOESM1_ESM.docx]

Supplementary Information for

**Transmission of stony coral tissue loss disease (SCTLD) in simulated ballast water confirms the potential for ship-born spread**

Michael S. Studivan,^1,2*^ Michelle Baptist,^2,3^ Vanessa Molina,^4^ Scott Riley,^4^ Matthew First,^5^ Nash Soderberg,^1,2^ Ewelina Rubin,^1,2†^ Ashley Rossin,^6^ Daniel M. Holstein,^6^ Ian C. Enochs^2^

^1^University of Miami

Cooperative Institute for Marine and Atmospheric Studies

4600 Rickenbacker Causeway

Miami, FL 33149

^2^NOAA Atlantic Oceanographic and Meteorological Laboratory

Ocean Chemistry and Ecosystems Division

4301 Rickenbacker Causeway

Miami, FL 33149

^3^University of Miami

Rosenstiel School of Marine and Atmospheric Science

4600 Rickenbacker Causeway

Miami, FL 33149

^4^Excet, Inc.

6225 Brandon Ave #360

Springfield, VA 22150

^5^U.S. Naval Research Laboratory

4555 Overlook Ave SW

Washington, DC 20375

^6^Louisiana State University

Department of Oceanography and Coastal Sciences

College of the Coast and Environment

Baton Rouge, LA 70803

*Corresponding author: studivanms@gmail.com

†Present address: University of Florida, 2033 Mowry Rd, Gainesville, FL 32611

**Keywords:** disease transmission, disease sources, ballast water management system, ballast water testing, histology, microbial communities

**Supplementary Methods**

UV biodosimetry testing and chamber selection

Prior to incorporation of UV treatment into the disease exposure apparatus described in the main text, we conducted a UV biodosimetry test to identify a suitable UV chamber based on ballast UV treatment requirements and experimental infrastructure capabilities. A UV collimated beam unit was designed and constructed by the Naval Research Lab, containing a low-pressure lamp with a wavelength of 254 nm within a UV lamp chamber from which UV emittance was measured beneath a 20 cm collimated cylinder housing. UV-based BWMS with Type Approval certification by the U.S. Coast Guard may use either medium-pressure lamps or low-pressure lamps (https://cgmix.uscg.mil). While medium-pressure lamps are generally more effective in ballast treatment systems, low-pressure lamps were used for bench-scale dosimetry and for experimental treatments due to lower heat generation (i.e., to avoid exposing corals to high temperatures) and ability to incorporate into existing experimental infrastructure.

To meet UV treatment requirements, a small-scale low-pressure lamp system was used to accommodate flow rates representative of the UV dosage used in a BWMS. A flow-through Sanitron S17A 3-GPM UV system (Atlantic Ultraviolet Corporation, Hauppauge, NY; Supplementary Fig. S1) was chosen, as it is specified for a maximum flow rate of 11.36 L min^-1^ (~3.0 gal min^-1^). To verify the fluence and efficacy of the UV system, the treatment response of the flow-through system was compared to the response of surrogate organism *Escherichia coli* using the controlled collimated beam. The fluence of the collimated beam was determined using a calibrated radiometer with incorporated correction factors, following standard protocols ^1^. Bacterial concentrations were determined using IDEXX unit dose SimPlates (IDEXX; Westbrook, ME) as described in detail below. Collimated beam tests were conducted for eight individual doses (0, 5, 10, 25, 50, 100, 200, and 300 mWs cm^-2^) to construct a standard curve of *E. coli* concentrations based on UV dose treatment. To estimate dosage using the Sanitron UV system, tests for four flow rates (0.5, 1.0, 2.0, and 3.0 gal min^-1^) were conducted and resulting bacterial concentrations were compared to those of the collimated beam standard curve. Flow rate range was chosen based on water flow capabilities for the experimental infrastructure and Sanitron UV system capabilities.

Ballast water analysis

For analyses requiring concentrated water samples, 5 L of water was collected from a runoff hose from each of the manifolds corresponding to water treatments in the UV experiment, or by using a submersible aquarium pump from each of the sealed ballast water containers in the ballast experiment. The 5 L water samples were subsequently filtered through 35-µm and 7-µm mesh sieves, where the concentrated sample retained on the 7-µm sieve (filtrand) was rinsed (using 0.22 µm-filtered seawater) into a sample container, and the volume was raised to 30–50 mL. For analyses not requiring concentration, 1 L of water was collected as described previously, but was not further processed prior to analysis.

For live cell counts, a 0.985 mL aliquot was removed from the concentrated sample and treated with CMFDA and FDA at 2.5 and 5 µM final concentrations, respectively, bringing the sample volume to 1 mL. Following a 10 min incubation period, the 1 mL sample was transferred onto a gridded Sedgewick Rafter (SR) counting chamber etched with 1 mm^2^ grids. A randomly predetermined number of squares within the slide were then scanned to detect living (i.e., fluorescing) organisms. Non-fluorescing organisms were classified as dead unless the object was actively moving. Organisms within the size class (as determined using microbeads with diameters approximately equal to the size thresholds) were categorized into general taxonomic groups and tallied. Concentrations of live organisms were calculated using Equation 1 where, *I* is the tally with the 5-mL aliquot (*A*, mL) and *C* and *S* are the volumes of the concentrated sample (mL) and the total sample (m^-3^), respectively. The sample dilution (*D*), if used, was incorporated into the equation as a non-dimensional value. For example, *D* would be 10 for a 10x dilution (i.e., 1 mL of sample combined with 9 mL of filtered water). The addition of CMFDA and FDA accounted for a small dilution (*D*, 1.015x) of the sample. Multivariate variation in live cell counts among broad taxonomic groups (i.e., absolute abundance) in each of the experiments was assessed using a two-way PERMANOVA in the package *vegan* ^2^. Pairwise PERMANOVA were conducted for significant factors using *pairwiseAdonis* ^3^, however, there were not enough permutations available (due to a limited dataset) to perform pairwise tests among time points and treatments for the ballast water experiment.

Equation 1. $P= \frac{ICD}{AS}$

To assess cellular physiology and health of phototrophs among treatments in both experiments, pulse amplitude modulation (PAM) variable fluorescence fluorometry (VFF) measurements were taken using a Water-PAM chlorophyll fluorometer (Waltz; Germany) of concentrated samples. Due to complications with the Water-PAM, however, VFF measurements were not collected until the second week of the UV experiment. A 3 mL sample was loaded into a quartz cuvette, placed into the detector, held for 10 s (to allow *F*_0_ readings to stabilize), and analyzed by starting the light excitation regime that generates estimates of initial fluorescence (*F*_0_) as a proximal measurement of algal concentrations, maximal fluorescence (*F*_M_), and photochemical yield (F_V_; (F_M_ – F_0_)/F_M_) as a proxy for the physiological health of phototrophs. VFF metrics F_0_  and F_V_ were analyzed from the respective experiments using a two-way ANOVA of Box-Cox transformed data, with Tukey’s pairwise tests of significant factors.

For quantification of heterotrophic bacteria using heterotrophic plate counts (HPC), IDEXX SimPlates use multi-enzyme substrates that produce a blue fluorescence when metabolized by waterborne bacteria. The sample and media were added to a SimPlate, incubated, and then examined for fluorescing wells. The number of fluorescing wells corresponds to a most probable number (MPN) of colony forming units (CFUs) in the original sample. The MPN values generated by the SimPlate HPC method correlate with the Pour Plate method using Total Plate Count Agar incubated at 35°C for 48 hours as described in Standard Methods for the Examination of Water and Wastewater ^4^. One-liter samples were diluted between 10^-2^ to 10^-4^ as needed to best reach a concentration range within plate limits of detection.

**Supplementary Results**

UV biodosimetry testing and chamber selection

The collimated beam standard curve ranged from bacterial concentrations of 2.4–73.8 CFUs mL^-1^, while concentrations in Sanitron UV-treated samples ranged from 2.0–16.1 CFU mL^-1^. Comparison to the collimated beam standard curve showed the Sanitron UV system flow rates of 0.5, 1.0, and 2.0 GPM were equivalent to ~300 mWs cm^-2^, while a flow rate of 11.36 L min^-1^ (~3.0 gal min^-1^) was equivalent to ~50 mWs cm^-2^ (Supplementary Fig. S1), which is considered acceptable for BWTS ^5^. Overall, for most treatment doses, flow-through *E. coli* concentrations decreased by ~10-fold in comparison to collimated beam concentrations at the same dose with the exception of 300 mWs cm^-2^.

Ballast water analysis

In the UV experiment, multivariate examination of live cell counts across broad taxonomic groups revealed no significant effect of treatments on communities, but there was a significant effect of sampling time point (PERMANOVA; Pseudo-*F*_3,35_ = 28.091, *p* < 0.001). Pairwise comparisons denoted significant differences between all time points except week 2 and week 3 (pairwise PERMANOVA; all *p* < 0.006; Supplementary Table S4). Waterborne microbial communities were generally dominated by dinoflagellates and pennate diatoms at the outset of the UV experiment, with a more than doubling in relative abundance of diatoms to >60% across all treatments through time (Supplementary Fig. S2). When collapsed into total live cell counts across all taxonomic groups, counts on week 1 (i.e., at the beginning of treatments) were an order of magnitude lower than all other weeks, with increases in abundance in subsequent weeks. Comparison of VFF metrics *F*_0_ and *F*_V_ identified treatment and sampling time point as significant factors affecting photochemical performance of waterborne microbes (all *p* < 0.001; Supplementary Fig. S2), with significant factor interactions. Overall, there was no notable trend in live organism concentrations or *F*_0_ values among treatments, however, *F*_V_ values varied above and below the healthy *F*_V_ threshold, indicating alternating cell health states through time.

In the ballast experiment, community cell counts were found to be significantly different among ballast water treatments and sampling time points, with a significant interaction effect (PERMANOVA; time: Pseudo-*F*_1,17_ = 2.976, *p* < 0.021; treatment: Pseudo-*F*_2,17_ = 4.071, *p* < 0.001; interaction: Pseudo-*F*_2,17_ = 2.729, *p* < 0.012; Supplementary Table S6). A similar community composition as in the UV experiment was observed, where dinoflagellates and pennate diatoms were initially more abundant, and diatom abundance increased in the following week of the experiment (Supplementary Fig. S3). The increase in diatom relative abundance, however, was more muted compared to the UV experiment, with the exception of the disease water ballasted for 120h, which showed an increase in abundance from ~25% to >70% with an additional seven days of ballasting. Similarly, VFF metrics *F*_0_ and *F*_V_ were both significantly different among treatments and through time with interactive effects (ANOVAs; all *p* < 0.001; Supplementary Table S6), though there were no observable patterns. *F*_V_ values were found to be above and below the healthy threshold through time and among treatments, indicating alternating health states as in the UV experiment (Supplementary Fig. S3).


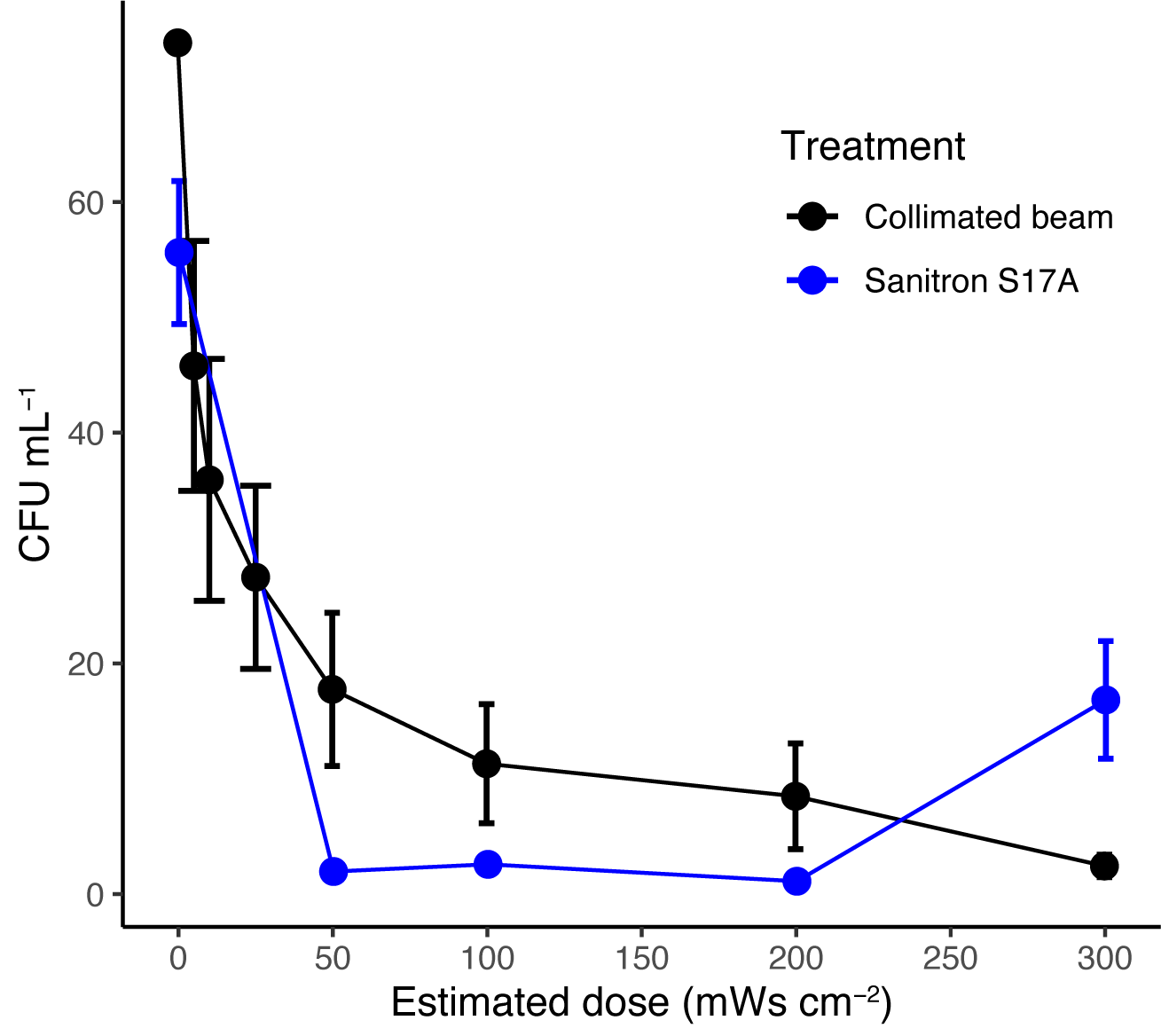


**Supplementary Figure S1. UV chamber validation.** Comparison of *E. coli* concentrations (colony forming units; CFUs mL^-1^) between collimated beam and Sanitron S17A UV treatments based on estimated dose.


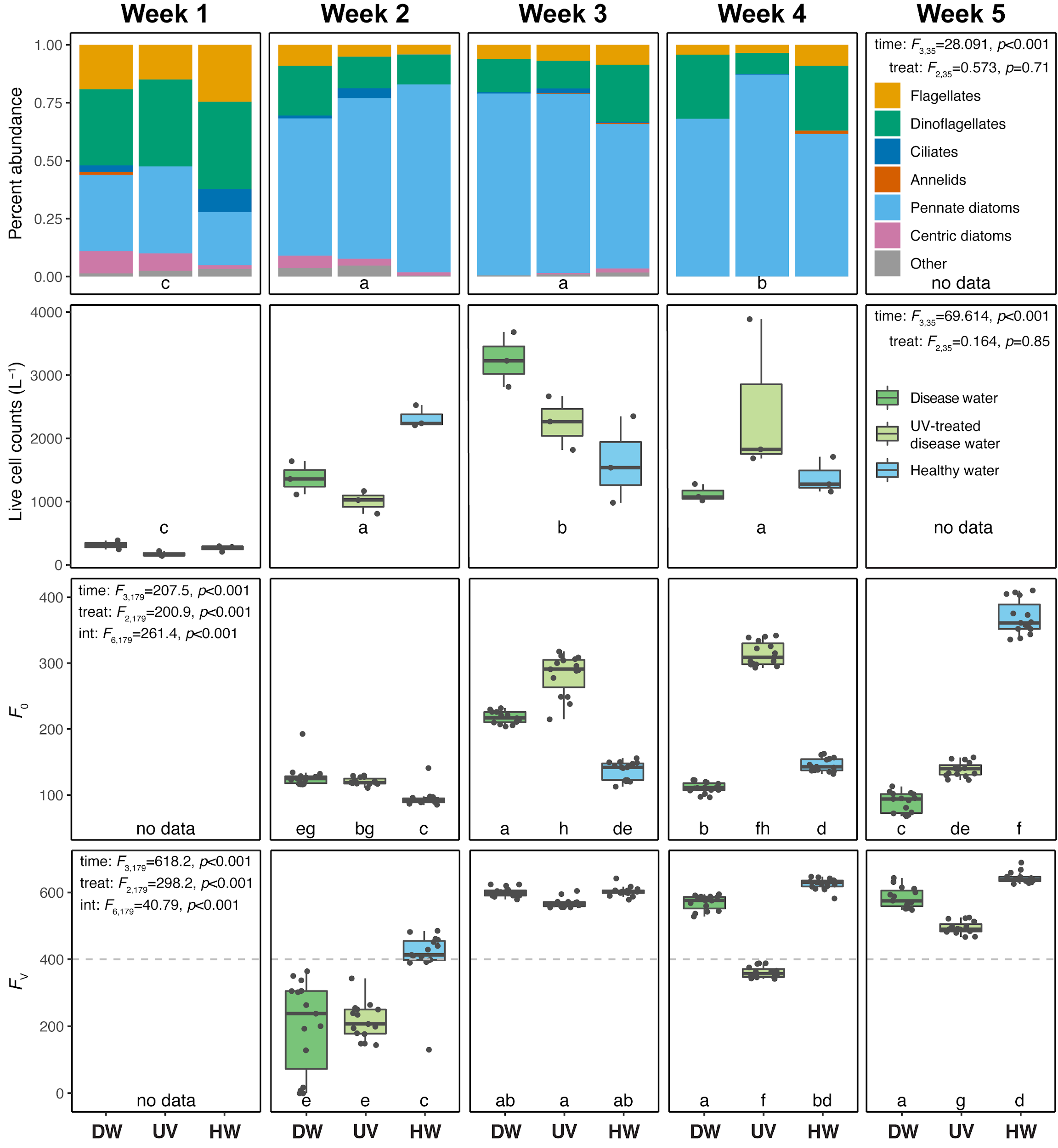


**Supplementary Figure S2. UV experiment water analysis.** (Top rows) Percent composition of live organisms measured by epifluorescent microscope counts, represented as broad taxonomic groups and total counts, across treatments and sampling time points. (Bottom rows) VFF F_0_ and F_V_ measurements for live organisms with a minimum dimension of ≥10 and <50 μm from the UV experiment. Error bars denote standard error of the mean, statistical outputs represent results of multivariate and univariate tests for each metric, and different letters denote significant differences among treatments and time points. Treatment abbreviations are as follows: disease water (DW), UV-treated disease water (UV), diseased coral direct contact (DC), and healthy water (HW).


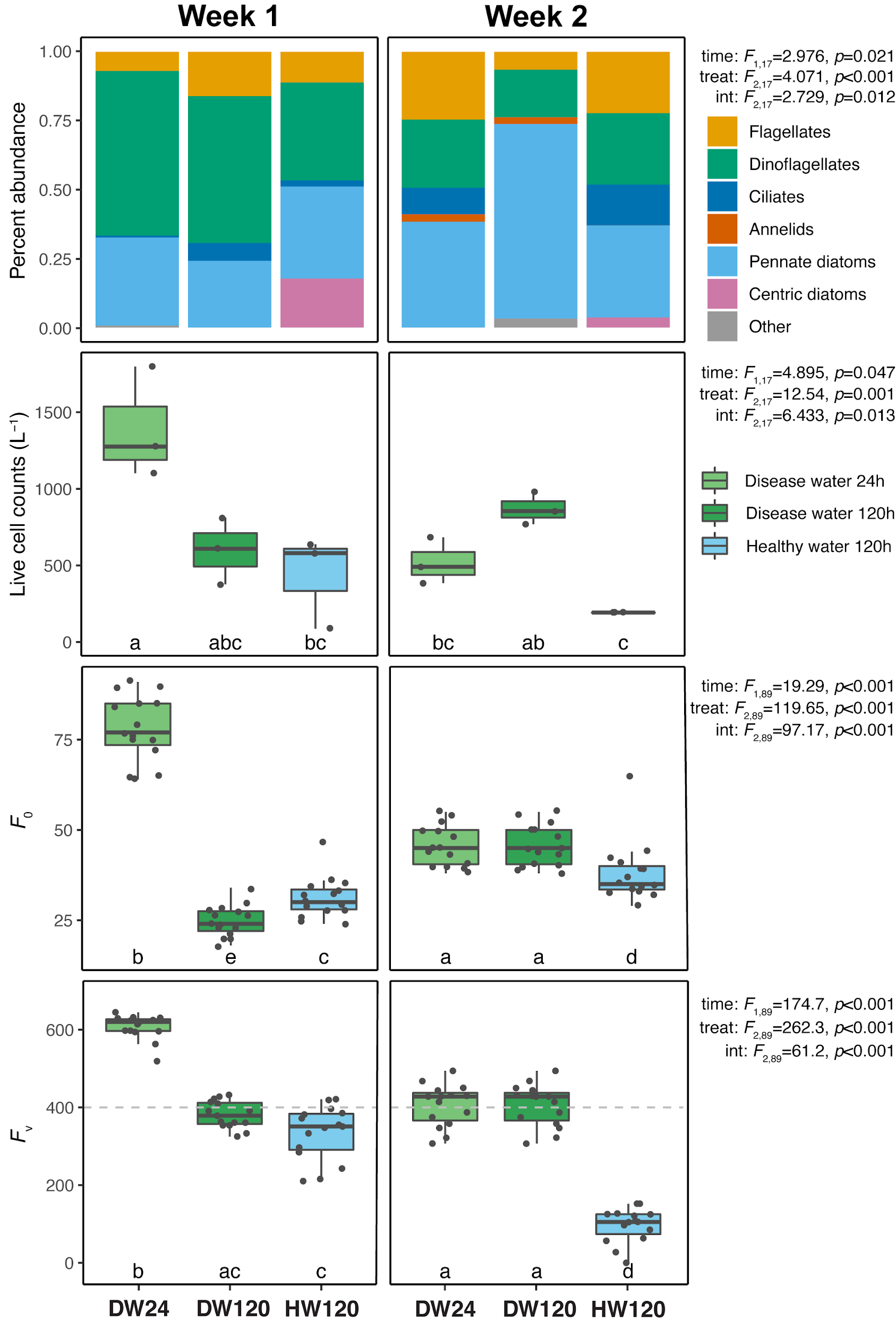


**Supplementary Figure S3. Ballast experiment water analysis.** (Top rows) Percent composition of live organisms measured by epifluorescent microscope counts, represented as broad taxonomic groups and total counts, across treatments and sampling time points. (Bottom rows) VFF F_0_ and F_V_ measurements for live organisms with a minimum dimension of ≥10 and <50 μm from the ballast experiment. Error bars denote standard error of the mean, statistical outputs represent results of multivariate and univariate tests for each metric, and different letters denote significant differences among treatments and time points. Treatment abbreviations are as follows: disease water 24h (DW24), disease water 120h (DW120), diseased coral direct contact (DC), and healthy water 120h (HW120).

**Supplementary Table S1. UV experiment test statistics.** Comparison of water dose required to elicit SCTLD signs among species and treatments. Non-significant *p* values reported as “ns,” and asterisk denotes significant interaction effect despite one model factor not being significant. Treatment abbreviations are as follows: disease water (DW), UV-treated disease water (UV), diseased coral direct contact (DC), and healthy water (HW).

| **Test** | **Comparison** | **df** | **Test Statistic** | ***p* Value** |
| --- | --- | --- | --- | --- |
| ANOVA | Species | 1,71 | 2.916 | ns |
|  | Treatment | 3,71 | 35.144 | < 0.001 |
|  | Species:Treatment | 2,71 | 2.999 | 0.0370* |
| Tukey's | Ps.DC – Of.DC |  |  | 0.022 |
|  | Of.DW – Of.DC |  |  | < 0.001 |
|  | Ps.DW – Of.DC |  |  | < 0.001 |
|  | Of.HW – Of.DC |  |  | < 0.001 |
|  | Ps.HW – Of.DC |  |  | < 0.001 |
|  | Of.UV – Of.DC |  |  | < 0.001 |
|  | Ps.UV – Of.DC |  |  | < 0.001 |
|  | Of.DW – Ps.DC |  |  | ns |
|  | Ps.DW – Ps.DC |  |  | ns |
|  | Of.HW – Ps.DC |  |  | < 0.001 |
|  | Ps.HW – Ps.DC |  |  | < 0.001 |
|  | Of.UV – Ps.DC |  |  | 0.003 |
|  | Ps.UV – Ps.DC |  |  | 0.013 |
|  | Ps.DW – Of.DW |  |  | ns |
|  | Of.HW – Of.DW |  |  | ns |
|  | Ps.HW – Of.DW |  |  | ns |
|  | Of.UV – Of.DW |  |  | ns |
|  | Ps.UV – Of.DW |  |  | ns |
|  | Of.HW – Ps.DW |  |  | ns |
|  | Ps.HW – Ps.DW |  |  | ns |
|  | Of.UV – Ps.DW |  |  | ns |
|  | Ps.UV – Ps.DW |  |  | ns |
|  | Ps.HW – Of.HW |  |  | ns |
|  | Of.UV – Of.HW |  |  | ns |
|  | Ps.UV – Of.HW |  |  | ns |
|  | Of.UV – Ps.HW |  |  | ns |
|  | Ps.UV – Ps.HW |  |  | ns |
|  | Ps.UV – Of.UV |  |  | ns |

**Supplementary Table S2. Ballast experiment test statistics.** Comparison of days to visible SCTLD signs among species and treatments. Non-significant *p* values reported as “ns,” and asterisk denotes significant interaction effect despite one model factor not being significant. Treatment abbreviations are as follows: disease water 24h (DW24), disease water 120h (DW120), diseased coral direct contact (DC), and healthy water 120h (HW120).

| **Test** | **Comparison** | **df** | **Test Statistic** | ***p* Value** |
| --- | --- | --- | --- | --- |
| ANOVA | Species | 1,36 | 0.011 | ns |
|  | Treatment | 3,36 | 7.449 | <0.001 |
|  | Species:Treatment | 1,36 | 15.408 | <0.001* |
| Tukey's | Ps.DC – Of.DC |  |  | 0.035 |
|  | Of.DW120 – Of.DC |  |  | <0.001 |
|  | Ps.DW120 – Of.DC |  |  | ns |
|  | Of.DW24 – Of.DC |  |  | 0.001 |
|  | Ps.DW24 – Of.DC |  |  |  |
|  | Of.HW120 – Of.DC |  |  | ns |
|  | Ps.HW120 – Of.DC |  |  |  |
|  | Of.DW120 – Ps.DC |  |  | ns |
|  | Ps.DW120 – Ps.DC |  |  | ns |
|  | Of.DW24 – Ps.DC |  |  | ns |
|  | Ps.DW24 – Ps.DC |  |  |  |
|  | Of.HW120 – Ps.DC |  |  | ns |
|  | Ps.HW120 – Ps.DC |  |  |  |
|  | Ps.DW120 – Of.DW120 |  |  | ns |
|  | Of.DW24 – Of.DW120 |  |  | ns |
|  | Ps.DW24 – Of.DW120 |  |  |  |
|  | Of.HW120 – Of.DW120 |  |  | ns |
|  | Ps.HW120 – Of.DW120 |  |  |  |
|  | Of.DW24 – Ps.DW120 |  |  | ns |
|  | Ps.DW24 – Ps.DW120 |  |  |  |
|  | Of.HW120 – Ps.DW120 |  |  | ns |
|  | Ps.HW120 – Ps.DW120 |  |  |  |
|  | Ps.DW24 – Of.DW24 |  |  |  |
|  | Of.HW120 – Of.DW24 |  |  | ns |
|  | Ps.HW120 – Of.DW24 |  |  |  |
|  | Of.HW120 – Ps.DW24 |  |  |  |
|  | Ps.HW120 – Ps.DW24 |  |  |  |
|  | Ps.HW120 – Of.HW120 |  |  |  |

**Supplementary Table S3. Histological analysis results.** Predictions of sample health status (healthy versus diseased) based on histological examination across treatments for the respective experiments, with the proportion of correct assignments. Treatment abbreviations for the UV experiment are as follows: disease water (DW), UV-treated disease water (UV), diseased coral direct contact (DC), and healthy water (HW). Treatment abbreviations for the ballast experiment are as follows: disease water 24h (DW24), disease water 120h (DW120), diseased coral direct contact (DC), and healthy water 120h (HW120).

| **Experiment** | **Treatment** | **Histological status** | | **Proportion correct (%)** |
| --- | --- | --- | --- | --- |
|  |  | **healthy** | **diseased** |  |
| UV | DW | 1 | 5 | 83% |
|  | UV | 0 | 6 | 100% |
|  | DC | 0 | 5 | 100% |
|  | HW | 4 | 3 | 57% |
| Ballast | DW-24 | 0 | 3 | 100% |
|  | DW-120 | 1 | 4 | 80% |
|  | DC | 0 | 6 | 100% |
|  | HW-120 | 1 | 3 | 25% |

**Supplementary Table S4. UV experiment water test statistics.** Analysis of water testing metrics (live cell counts and VFF metrics *F*_0_ and *F*_V_) among sampling time points and treatments. Non-significant p values reported as “ns,” and asterisk denotes significant interaction effect despite one model factor not being significant. Treatment abbreviations are as follows: disease water (DW), UV-treated disease water (UV), diseased coral direct contact (DC), and healthy water (HW).

| **Dataset** | **Test** | **Comparison** | **df** | **Test Statistic** | ***p* Value** |
| --- | --- | --- | --- | --- | --- |
| Cell counts | PERMANOVA | Time | 3,35 | 28.091 | 0.001 |
| community |  | Treatment | 2,35 | 0.5732 | ns |
|  |  | Time:Treatment | 6,35 | 3.488 | <0.001* |
|  | pairwise | Week 1 – Week 2 | 1,17 | 25.279 | 0.001 |
|  | PERMANOVA | Week 1 – Week 3 | 1,17 | 33.668 | 0.001 |
|  |  | Week 1 – Week 4 | 1,17 | 36.01 | 0.001 |
|  |  | Week 2 – Week 3 | 1,17 | 3.025 | ns |
|  |  | Week 2 – Week 4 | 1,17 | 6.571 | 0.001 |
|  |  | Week 3 – Week 4 | 1,17 | 4.501 | 0.006 |
| Cell counts | ANOVA | Time | 3,35 | 69.614 | <0.001 |
| total |  | Treatment | 2,35 | 0.164 | ns |
|  |  | Time:Treatment | 6,35 | 8.532 | <0.001 |
|  | Tukey | Week 1 – Week 2 |  |  | <0.001 |
|  |  | Week 1 – Week 3 |  |  | <0.001 |
|  |  | Week 1 – Week 4 |  |  | <0.001 |
|  |  | Week 2 – Week 3 |  |  | 0.004 |
|  |  | Week 2 – Week 4 |  |  | ns |
|  |  | Week 3 – Week 4 |  |  | 0.001 |
| VFF *F*_0_ | ANOVA | Time | 3,179 | 207.5 | <0.001 |
|  |  | Treatment | 2,179 | 200.9 | <0.001 |
|  |  | Time:Treatment | 6,179 | 261.4 | <0.001 |
|  | Tukey | Week 3.DW – Week 2.DW |  |  | <0.001 |
|  |  | Week 4.DW – Week 2.DW |  |  | 0.006 |
|  |  | Week 6.DW – Week 2.DW |  |  | <0.001 |
|  |  | Week 2.HW – Week 2.DW |  |  | <0.001 |
|  |  | Week 3.HW – Week 2.DW |  |  | ns |
|  |  | Week 4.HW – Week 2.DW |  |  | 0.024 |
|  |  | Week 6.HW – Week 2.DW |  |  | <0.001 |
|  |  | Week 2.UV – Week 2.DW |  |  | ns |
|  |  | Week 3.UV – Week 2.DW |  |  | <0.001 |
|  |  | Week 4.UV – Week 2.DW |  |  | <0.001 |
|  |  | Week 6.UV – Week 2.DW |  |  | ns |
|  |  | Week 4.DW – Week 3.DW |  |  | <0.001 |
|  |  | Week 6.DW – Week 3.DW |  |  | <0.001 |
|  |  | Week 2.HW – Week 3.DW |  |  | <0.001 |
|  |  | Week 3.HW – Week 3.DW |  |  | <0.001 |
|  |  | Week 4.HW – Week 3.DW |  |  | <0.001 |
|  |  | Week 6.HW – Week 3.DW |  |  | <0.001 |
|  |  | Week 2.UV – Week 3.DW |  |  | <0.001 |
|  |  | Week 3.UV – Week 3.DW |  |  | <0.001 |
|  |  | Week 4.UV – Week 3.DW |  |  | <0.001 |
|  |  | Week 6.UV – Week 3.DW |  |  | <0.001 |
|  |  | Week 6.DW – Week 4.DW |  |  | <0.001 |
|  |  | Week 2.HW – Week 4.DW |  |  | <0.001 |
|  |  | Week 3.HW – Week 4.DW |  |  | <0.001 |
|  |  | Week 4.HW – Week 4.DW |  |  | <0.001 |
|  |  | Week 6.HW – Week 4.DW |  |  | <0.001 |
|  |  | Week 2.UV – Week 4.DW |  |  | ns |
|  |  | Week 3.UV – Week 4.DW |  |  | <0.001 |
|  |  | Week 4.UV – Week 4.DW |  |  | <0.001 |
|  |  | Week 6.UV – Week 4.DW |  |  | <0.001 |
|  |  | Week 2.HW – Week 6.DW |  |  | ns |
|  |  | Week 3.HW – Week 6.DW |  |  | <0.001 |
|  |  | Week 4.HW – Week 6.DW |  |  | <0.001 |
|  |  | Week 6.HW – Week 6.DW |  |  | <0.001 |
|  |  | Week 2.UV – Week 6.DW |  |  | <0.001 |
|  |  | Week 3.UV – Week 6.DW |  |  | <0.001 |
|  |  | Week 4.UV – Week 6.DW |  |  | <0.001 |
|  |  | Week 6.UV – Week 6.DW |  |  | <0.001 |
|  |  | Week 3.HW – Week 2.HW |  |  | <0.001 |
|  |  | Week 4.HW – Week 2.HW |  |  | <0.001 |
|  |  | Week 6.HW – Week 2.HW |  |  | <0.001 |
|  |  | Week 2.UV – Week 2.HW |  |  | <0.001 |
|  |  | Week 3.UV – Week 2.HW |  |  | <0.001 |
|  |  | Week 4.UV – Week 2.HW |  |  | <0.001 |
|  |  | Week 6.UV – Week 2.HW |  |  | <0.001 |
|  |  | Week 4.HW – Week 3.HW |  |  | ns |
|  |  | Week 6.HW – Week 3.HW |  |  | <0.001 |
|  |  | Week 2.UV – Week 3.HW |  |  | 0.038 |
|  |  | Week 3.UV – Week 3.HW |  |  | <0.001 |
|  |  | Week 4.UV – Week 3.HW |  |  | <0.001 |
|  |  | Week 6.UV – Week 3.HW |  |  | ns |
|  |  | Week 6.HW – Week 4.HW |  |  | <0.001 |
|  |  | Week 2.UV – Week 4.HW |  |  | <0.001 |
|  |  | Week 3.UV – Week 4.HW |  |  | <0.001 |
|  |  | Week 4.UV – Week 4.HW |  |  | <0.001 |
|  |  | Week 6.UV – Week 4.HW |  |  | ns |
|  |  | Week 2.UV – Week 6.HW |  |  | <0.001 |
|  |  | Week 3.UV – Week 6.HW |  |  | 0.004 |
|  |  | Week 4.UV – Week 6.HW |  |  | ns |
|  |  | Week 6.UV – Week 6.HW |  |  | <0.001 |
|  |  | Week 3.UV – Week 2.UV |  |  | <0.001 |
|  |  | Week 4.UV – Week 2.UV |  |  | <0.001 |
|  |  | Week 6.UV – Week 2.UV |  |  | 0.015 |
|  |  | Week 4.UV – Week 3.UV |  |  | ns |
|  |  | Week 6.UV – Week 3.UV |  |  | <0.001 |
|  |  | Week 6.UV – Week 4.UV |  |  | <0.001 |
| VFF *F*_V_ | ANOVA | Time | 3,179 | 618.2 | <0.001 |
|  |  | Treatment | 2,179 | 298.2 | <0.001 |
|  |  | Time:Treatment | 6,179 | 40.79 | <0.001 |
|  | Tukey | Week 3.DW – Week 2.DW |  |  | <0.001 |
|  |  | Week 4.DW – Week 2.DW |  |  | <0.001 |
|  |  | Week 6.DW – Week 2.DW |  |  | <0.001 |
|  |  | Week 2.HW – Week 2.DW |  |  | <0.001 |
|  |  | Week 3.HW – Week 2.DW |  |  | <0.001 |
|  |  | Week 4.HW – Week 2.DW |  |  | <0.001 |
|  |  | Week 6.HW – Week 2.DW |  |  | <0.001 |
|  |  | Week 2.UV – Week 2.DW |  |  | ns |
|  |  | Week 3.UV – Week 2.DW |  |  | <0.001 |
|  |  | Week 4.UV – Week 2.DW |  |  | <0.001 |
|  |  | Week 6.UV – Week 2.DW |  |  | <0.001 |
|  |  | Week 4.DW – Week 3.DW |  |  | ns |
|  |  | Week 6.DW – Week 3.DW |  |  | ns |
|  |  | Week 2.HW – Week 3.DW |  |  | <0.001 |
|  |  | Week 3.HW – Week 3.DW |  |  | ns |
|  |  | Week 4.HW – Week 3.DW |  |  | ns |
|  |  | Week 6.HW – Week 3.DW |  |  | 0.008 |
|  |  | Week 2.UV – Week 3.DW |  |  | <0.001 |
|  |  | Week 3.UV – Week 3.DW |  |  | ns |
|  |  | Week 4.UV – Week 3.DW |  |  | <0.001 |
|  |  | Week 6.UV – Week 3.DW |  |  | <0.001 |
|  |  | Week 6.DW – Week 4.DW |  |  | ns |
|  |  | Week 2.HW – Week 4.DW |  |  | <0.001 |
|  |  | Week 3.HW – Week 4.DW |  |  | ns |
|  |  | Week 4.HW – Week 4.DW |  |  | <0.001 |
|  |  | Week 6.HW – Week 4.DW |  |  | <0.001 |
|  |  | Week 2.UV – Week 4.DW |  |  | <0.001 |
|  |  | Week 3.UV – Week 4.DW |  |  | ns |
|  |  | Week 4.UV – Week 4.DW |  |  | <0.001 |
|  |  | Week 6.UV – Week 4.DW |  |  | <0.001 |
|  |  | Week 2.HW – Week 6.DW |  |  | <0.001 |
|  |  | Week 3.HW – Week 6.DW |  |  | ns |
|  |  | Week 4.HW – Week 6.DW |  |  | 0.018 |
|  |  | Week 6.HW – Week 6.DW |  |  | <0.001 |
|  |  | Week 2.UV – Week 6.DW |  |  | <0.001 |
|  |  | Week 3.UV – Week 6.DW |  |  | ns |
|  |  | Week 4.UV – Week 6.DW |  |  | <0.001 |
|  |  | Week 6.UV – Week 6.DW |  |  | <0.001 |
|  |  | Week 3.HW – Week 2.HW |  |  | <0.001 |
|  |  | Week 4.HW – Week 2.HW |  |  | <0.001 |
|  |  | Week 6.HW – Week 2.HW |  |  | <0.001 |
|  |  | Week 2.UV – Week 2.HW |  |  | <0.001 |
|  |  | Week 3.UV – Week 2.HW |  |  | <0.001 |
|  |  | Week 4.UV – Week 2.HW |  |  | 0.016 |
|  |  | Week 6.UV – Week 2.HW |  |  | <0.001 |
|  |  | Week 4.HW – Week 3.HW |  |  | ns |
|  |  | Week 6.HW – Week 3.HW |  |  | 0.016 |
|  |  | Week 2.UV – Week 3.HW |  |  | <0.001 |
|  |  | Week 3.UV – Week 3.HW |  |  | ns |
|  |  | Week 4.UV – Week 3.HW |  |  | <0.001 |
|  |  | Week 6.UV – Week 3.HW |  |  | <0.001 |
|  |  | Week 6.HW – Week 4.HW |  |  | ns |
|  |  | Week 2.UV – Week 4.HW |  |  | <0.001 |
|  |  | Week 3.UV – Week 4.HW |  |  | <0.001 |
|  |  | Week 4.UV – Week 4.HW |  |  | <0.001 |
|  |  | Week 6.UV – Week 4.HW |  |  | <0.001 |
|  |  | Week 2.UV – Week 6.HW |  |  | <0.001 |
|  |  | Week 3.UV – Week 6.HW |  |  | <0.001 |
|  |  | Week 4.UV – Week 6.HW |  |  | <0.001 |
|  |  | Week 6.UV – Week 6.HW |  |  | <0.001 |
|  |  | Week 3.UV – Week 2.UV |  |  | <0.001 |
|  |  | Week 4.UV – Week 2.UV |  |  | <0.001 |
|  |  | Week 6.UV – Week 2.UV |  |  | <0.001 |
|  |  | Week 4.UV – Week 3.UV |  |  | <0.001 |
|  |  | Week 6.UV – Week 3.UV |  |  | <0.001 |
|  |  | Week 6.UV – Week 4.UV |  |  | <0.001 |

**Supplementary Table S5. UV experiment water bacterial counts.** Most probable number (MPN) estimates of heterotrophic bacteria colony forming units (CFUs) across sampling time points and treatments, including 95% CI. Treatment abbreviations are as follows: disease water (DW), UV-treated disease water (UV), diseased coral direct contact (DC), and healthy water (HW).

| **Sampling Time Point** | **DW MPN (CFU mL^-1^)** | **95% Confidence** | | **UV MPN (CFU mL^-1^)** | **95% Confidence** | | | **HW MPN (CFU mL^-1^)** | | **95% Confidence** | | | | |
| --- | --- | --- | --- | --- | --- | --- | --- | --- | --- | --- | --- | --- | --- | --- |
|  |  | **Lower** | **Upper** |  | **Lower** | | **Upper** |  |  | **Lower** | | | **Upper** | |
| Week 1 (8-Jul-21) | 1.8 x 10^3^ | 9.6 x 10^2^ | 3.5 x 10^3^ | 3.8 x 10^3^ | 9.0 x 10^3^ | 6.4 x 10^3^ | | | 9.0 x 10^3^ | | 2.4 x 10^3^ | 6.1 x 10^3^ | |  |
| Week 2 (14-Jul-21) | ≤6.0 x 10^2^ | ≤2.1 x 10^2^ | ≤1.9 x 10^3^ | 8.0 x 10^2^ | 2.7 x 10^3^ | 1.6 x 10^3^ | | | 2.7 x 10^3^ | | 3.0 x 10^2^ | 2.2 x 10^3^ | |  |
| Week 3 (21-Jul-21) | 1.0 x 10^3^ | 7.8 x 10^2^ | 1.4 x 10^3^ | 3.1 x 10^2^ | 4.6 x 10^2^ | 3.0 x 10^2^ | | | 4.6 x 10^2^ | | 1.9 x 10^2^ | 5.2 x 10^2^ | |  |
| Week 4 (28-Jul-21) | >7.3 x 10^4^ | >4.7 x 10^4^ | >1.1 x 10^5^ | ≥6.3 x 10^4^ | 5.2 x 10^4^ | 3.8 x 10^4^ | | | 5.2 x 10^4^ | | ≥4.2 x 10^4^ | ≥9.6 x 10^4^ | |  |
| Week 6 (11-Aug-21) | >8.8 x 10^3^ | >6.3 x 10^3^ | >1.2 x 10^4^ | ≥2.5 x 10^4^ | 1.7 x 10^4^ | 1.2 x 10^4^ | | | 1.7 x 10^4^ | | ≥1.9 x 10^4^ | ≥3.3 x 10^4^ | |  |

**Supplementary Table S6. Ballast experiment water test statistics.** Analysis of water testing metrics (live cell counts and VFF metrics *F*_0_ and *F*_V_) among sampling time points and treatments. Non-significant p values reported as “ns.” Treatment abbreviations are as follows: disease water 24h (DW24), disease water 120h (DW120), diseased coral direct contact (DC), and healthy water 120h (HW120).

| **Dataset** | **Test** | **Comparison** | **df** | **Test Statistic** | ***p* Value** |
| --- | --- | --- | --- | --- | --- |
| Cell counts | PERMANOVA | Time | 1,17 | 2.976 | 0.021 |
| community |  | Treatment | 2,17 | 4.071 | <0.001 |
|  |  | Time:Treatment | 2,17 | 2.729 | 0.012 |
| Cell counts | ANOVA | Time | 1,17 | 4.895 | 0.047 |
| total |  | Treatment | 2,17 | 12.540 | 0.001 |
|  |  | Time:Treatment | 2,17 | 6.433 | 0.013 |
|  | Tukey | Week 2.DW120 – Week 1.DW120 |  |  | ns |
|  |  | Week 1.DW24 – Week 1.DW120 |  |  | ns |
|  |  | Week 2.DW24 – Week 1.DW120 |  |  | ns |
|  |  | Week 1.HW120 – Week 1.DW120 |  |  | ns |
|  |  | Week 2.HW120 – Week 1.DW120 |  |  | ns |
|  |  | Week 1.DW24 – Week 2.DW120 |  |  | ns |
|  |  | Week 2.DW24 – Week 2.DW120 |  |  | ns |
|  |  | Week 1.HW120 – Week 2.DW120 |  |  | ns |
|  |  | Week 2.HW120 – Week 2.DW120 |  |  | 0.017 |
|  |  | Week 2.DW24 – Week 1.DW24 |  |  | 0.027 |
|  |  | Week 1.HW120 – Week 1.DW24 |  |  | 0.007 |
|  |  | Week 2.HW120 – Week 1.DW24 |  |  | <0.001 |
|  |  | Week 1.HW120 – Week 2.DW24 |  |  | ns |
|  |  | Week 2.HW120 – Week 2.DW24 |  |  | ns |
|  |  | Week 2.HW120 – Week 1.HW120 |  |  | ns |
| VFF *F*_0_ | ANOVA | Time | 1,89 | 19.29 | <0.001 |
|  |  | Treatment | 2,89 | 119.65 | <0.001 |
|  |  | Time:Treatment | 2,89 | 97.17 | <0.001 |
|  | Tukey | Week 2.DW120 – Week 1.DW120 |  |  | <0.001 |
|  |  | Week 1.DW24 – Week 1.DW120 |  |  | <0.001 |
|  |  | Week 2.DW24 – Week 1.DW120 |  |  | <0.001 |
|  |  | Week 1.HW120 – Week 1.DW120 |  |  | <0.001 |
|  |  | Week 2.HW120 – Week 1.DW120 |  |  | <0.001 |
|  |  | Week 1.DW24 – Week 2.DW120 |  |  | <0.001 |
|  |  | Week 2.DW24 – Week 2.DW120 |  |  | ns |
|  |  | Week 1.HW120 – Week 2.DW120 |  |  | <0.001 |
|  |  | Week 2.HW120 – Week 2.DW120 |  |  | 0.015 |
|  |  | Week 2.DW24 – Week 1.DW24 |  |  | <0.001 |
|  |  | Week 1.HW120 – Week 1.DW24 |  |  | <0.001 |
|  |  | Week 2.HW120 – Week 1.DW24 |  |  | <0.001 |
|  |  | Week 1.HW120 – Week 2.DW24 |  |  | <0.001 |
|  |  | Week 2.HW120 – Week 2.DW24 |  |  | 0.015 |
|  |  | Week 2.HW120 – Week 1.HW120 |  |  | 0.006 |
| VFF *F*_V_ | ANOVA | Time | 1,89 | 174.7 | <0.001 |
|  |  | Treatment | 2,89 | 262.3 | <0.001 |
|  |  | Time:Treatment | 2,89 | 61.2 | <0.001 |
|  | Tukey | Week 2.DW120 – Week 1.DW120 |  |  | ns |
|  |  | Week 1.DW24 – Week 1.DW120 |  |  | <0.001 |
|  |  | Week 2.DW24 – Week 1.DW120 |  |  | ns |
|  |  | Week 1.HW120 – Week 1.DW120 |  |  | ns |
|  |  | Week 2.HW120 – Week 1.DW120 |  |  | <0.001 |
|  |  | Week 1.DW24 – Week 2.DW120 |  |  | <0.001 |
|  |  | Week 2.DW24 – Week 2.DW120 |  |  | ns |
|  |  | Week 1.HW120 – Week 2.DW120 |  |  | 0.002 |
|  |  | Week 2.HW120 – Week 2.DW120 |  |  | <0.001 |
|  |  | Week 2.DW24 – Week 1.DW24 |  |  | <0.001 |
|  |  | Week 1.HW120 – Week 1.DW24 |  |  | <0.001 |
|  |  | Week 2.HW120 – Week 1.DW24 |  |  | <0.001 |
|  |  | Week 1.HW120 – Week 2.DW24 |  |  | 0.002 |
|  |  | Week 2.HW120 – Week 2.DW24 |  |  | <0.001 |
|  |  | Week 2.HW120 – Week 1.HW120 |  |  | <0.001 |

**Supplementary Table S7. Ballast experiment water bacterial counts.** Most probable number (MPN) estimates of heterotrophic bacteria colony forming units (CFUs) across sampling time points and treatments, including 95% CI. Treatment abbreviations are as follows: disease water 24h (DW24), disease water 120h (DW120), diseased coral direct contact (DC), and healthy water 120h (HW120).

| **Sampling Time Point** | **DW24 MPN (CFU mL^-1^)** | **95% Confidence** | | **DW120 MPN (CFU mL^-1^)** | **95% Confidence** | | **HW120 MPN (CFU mL^-1^)** | **95% Confidence** | |
| --- | --- | --- | --- | --- | --- | --- | --- | --- | --- |
|  |  | **Lower** | **Upper** |  | **Lower** | **Upper** |  | **Lower** | **Upper** |
| Week 1 (28-Jul-21) | 5.0 x 10^5^ | 3.6 x 10^5^ | 6.9 x 10^5^ | 5.6 x 10^5^ | 3.9 x 10^5^ | 8.1 x 10^5^ | 6.1 x 10^5^ | ≥4.1 x 10^5^ | ≥8.9 x 10^5^ |
| Week 2 (4-Aug-21) | ≥7.3 x 10^5^ | ≥4.7 x 10^5^ | ≥1.1 x 10^6^ | ≥6.2 x 10^5^ | ≥4.2 x 10^5^ | ≥9.1 x 10^5^ | >7.3 x 10^5^ | >4.7 x 10^5^ | >1.1 x 10^6^ |

**References**

1. Bolton, J. R. & Linden, K. G. Standardization of methods for fluence (UV dose) determination in bench-scale UV experiments. *Journal of Environmental Engineering* **129**, 209–215 (2003).

2. Oksanen, J. *et al.* *vegan*: community ecology package. *R* package version 2.0-10. (2015).

3. Martinez Arbizu, P. *pairwiseAdonis*: Pairwise multilevel comparison using adonis. *R* package version 0.4. (2020).

4. Water Environmental Federation & American Public Health Association. Standard methods for the examination of water and wastewater. *Washington, D.C.* 21 (2005).

5. U.S. Environmental Protection Agency. Generic protocol for the verification of ballast water treatment technology, version 5.1. Report number EPA/600/R-10/146. *Washington, D.C.* 157 (2010).
